# Existence, uniqueness and comparison results for BSDEs with Lévy jumps in an extended monotonic generator setting

**DOI:** 10.1186/s41546-018-0034-y

**Published:** 2018-12-28

**Authors:** Christel Geiss, Alexander Steinicke

**Affiliations:** 1 University of Jyvaskyla, Department of Mathematics and Statistics, P.O. Box 35, Jyvaskyla, 40014 Finland; 2 Department of Mathematics and Information Technology, Montanuniversitaet Leoben, Leoben, Austria

**Keywords:** Backward stochastic differential equation, Lévy process, comparison theorem, existence and uniqueness, 60H10

## Abstract

We show that the comparison results for a backward SDE with jumps established in Royer (Stoch. Process. Appl 116: 1358–1376, 2006) and Yin and Mao (J. Math. Anal. Appl 346: 345–358, 2008) hold under more simplified conditions. Moreover, we prove existence and uniqueness allowing the coefficients in the linear growth- and monotonicity-condition for the generator to be random and time-dependent. In the *L*^2^-case with linear growth, this also generalizes the results of Kruse and Popier (Stochastics 88: 491–539, 2016). For the proof of the comparison result, we introduce an approximation technique: Given a BSDE driven by Brownian motion and Poisson random measure, we approximate it by BSDEs where the Poisson random measure admits only jumps of size larger than 1/*n*.

## Introduction

In this paper, we study backward stochastic differential equations (BSDEs) of the form

1 $$ Y_{t}=\xi+{\int}_{t}^{T} f\left(s,Y_{s},Z_{s},U_{s}\right)ds-{\int}_{t}^{T} Z_{s} {dW}_{s}-{\int}_{{]t,T]}\times(\mathbb{R}\setminus\{0\})}U_{s}(x)\tilde{N}(ds,dx),  $$

where *W* denotes a one-dimensional Brownian motion and $\tilde {N}$ a compensated Poisson random measure belonging to a given Lévy process with Lévy measure *ν*. In particular, our focus lies on comparison results and existence and uniqueness of solutions.

Comparison theorems state that—under certain conditions—if *ξ*≤*ξ*^′^ and *f*≤*f*^′^, then the process *Y* of the solution satisfies *Y*_*t*_≤*Yt*′ for all *t*∈[0,*T*]. These types of theorems in the case of one-dimensional, Brownian BSDEs has been treated by Peng (1992), El Karoui et al. (1997, 2009), and Cao and Yan (1999).

In (Barles et al. (1997), Remark 2.7) a counterexample was given, which shows that in the jump case the conditions *ξ*≤*ξ*^′^ and *f*≤*f*^′^ are not sufficient to guarantee *Y*≤*Y*^′^. They propose an additional sufficient condition which has been generalized by Kruse and Popier (2016), Royer (2006), Yin and Mao (2008), Becherer et al. (2018) (allowing more general jump processes), and Cohen et al. (2010) (for BSDEs driven by martingales). The condition of Kruse and Popier (2016) reads (in our *L*^2^-setting) as follows: for each $s,y,z,u, u^{\prime } \in [0,T]\times \mathbb {R} \times \mathbb {R} \times L^{2}(\nu)\times L^{2}(\nu)$ there is a progressively measurable process $\gamma ^{y,z,u,u{\prime }}\colon \Omega \times {[0,T]}\times \mathbb {R}\setminus \{0\}\to \mathbb {R}$ such that

2 $$\begin{array}{@{}rcl@{}}  && f(s,y,z,u)-f\left(s,y,z,u^{\prime}\right)\leq \int_{\mathbb{R}\setminus\{0\}}\left(u(x)-u^{\prime}(x)\right)\gamma_{s}^{y,z,u,u{\prime}}(x)\nu(dx),  \\ && -1 \leq \gamma^{y,z,u,u{\prime}}_{s}(x) \quad \text{and} \quad \sup_{s,\omega, y,z,u,u{\prime}} \left|\gamma^{y,z,u,u{\prime}}_{s}\right| \in L^{2}(\nu). \end{array} $$

One of the main results in the present paper is Theorem 3.5 which states that (2) can be replaced by the simpler condition

3 $$\begin{array}{@{}rcl@{}}  &&f(s,y,z,u)- f\left(s,y,z,u^{\prime}\right)\leq {\int}_{\mathbb{R}\setminus\{0\}}\left(u^{\prime}(x)-u(x)\right)\nu(dx), \quad \mathbb{P}\otimes\lambda\text{-a.e.}  \\ &&\text{for all} \,\, u, u^{\prime}\in L^{2}(\nu) \, \text{ with} \, u\leq u^{\prime}. \end{array} $$

Notice that the r.h.s. is infinite for *u*^′^(*x*)−*u*(*x*)∉*L*^1^(*ν*). Clearly, (3) is a weaker condition than (2), because one only needs to check the inequality for those *u*,*u*^′^∈*L*^2^(*ν*) for which *u*≤*u*^′^ holds. Moreover, we do not need any *L*^2^(*ν*) condition for $\gamma ^{y,z,u,u{\prime }}_{s}$ but we choose $\gamma ^{y,z,u,u{\prime }}_{s}(x)=-1.$ Under the constraint $ -1 \leq \gamma ^{y,z,u,u{\prime }}_{s}(x),$ the choice $\gamma ^{y,z,u,u{\prime }}_{s}(x)=-1$ yields for *u*^′^−*u*≥0 the largest possible expression on the r.h.s. of (2), so that (3) can be seen as the weakest possible condition which (2) could impose on *f*.

For a finite Lévy measure *ν*, Theorem 3.5 can be shown using only elementary means.

Another main result is a method of how to approximate a BSDE driven by a Lévy process with an infinite measure *ν*, by a sequence of BSDEs where the driving processes have a finite Lévy measure. We apply this result to show the comparison theorem for BSDEs driven by a general Lévy process. The proof relies on the Jankov–von Neumann theorem on measurable sections/uniformizations (this theorem is also important for dynamic programming, see El Karoui and Tan (2013). Under certain conditions on the generator, the approximating solutions can be interpreted as nonlinear conditional expectations (in the sense of Peng (2010)), conditioned on a Lévy process whose jumps are not of arbitrarily small size. (See the comments after Theorem 3.4.)

Studying the existence, uniqueness, and comparison results by Darling and Pardoux (1997), Pardoux and Zhang (1996), Pardoux (1997), Fan and Jiang (2012), Royer (2006), Situ (1997), Yin and Mao (2008), Kruse and Popier (2016, 2017), Yao (2017), and Sow (2014), one notices that one can unify and generalize the assumptions on *f*.

Indeed, and this is our third main result, in the case of *L*^2^-solutions, for a progressively measurable generator *f* with linear growth, it suffices to assume (cf. Theorems 3.1 and 3.5) the following growth- and monotonicity conditions with time-dependent, random coefficients:

- |*f*(*ω*,*s*,*y*,*z*,*u*)|≤*F*(*s*,*ω*)+*K*_1_(*s*,*ω*)|*y*|+*K*_2_(*s*,*ω*)(|*z*|+∥*u*∥),
- (*y*−*y*^′^)(*f*_1_(*ω*,*s*,*y*,*z*,*u*)−*f*_1_(*ω*,*s*,*y*^′^,*z*^′^,*u*^′^))
  ≤*α*(*s*)*ρ*(|*y*−*y*^′^|^2^)+*β*(*s*,*ω*)|*y*−*y*^′^|(|*z*−*z*^′^|+∥*u*−*u*^′^∥),

with *α*∈*L*^1^([0,*T*]) and *F* being nonnegative and progressively measurable such that $\mathbb {E}\left [ \left (\int _{0}^{T} F(\omega, t)dt \right)^{2}\right ] < \infty.$ The processes *K*_1_,*K*_2_, and *β* are nonnegative and progressively measurable such that for a constant *c*>0,

$${\int}_{0}^{T}\left(K_{1}(s)+K_{2}(s)^{2}+\beta(s)^{2}\right)ds < c,\quad\mathbb{P}\text{-a.s.}$$

The concave function *ρ* in the monotonicity condition may grow faster than linear at zero and satisfies $\int _{0^{+}} 1/\rho (x)dx=\infty.$ This type of function already appeared in context with BSDEs in Mao (1995) in 1997.

These assumptions also extend the monotonicity condition of Kruse and Popier (2016, 2017), for the *L*^2^-case with linear growth, since the coefficients in our setting take randomness, the function *ρ* and time-dependence into account. BSDEs with time-dependent coefficients appear, for example, in Gobet and Turkedjiev (2016).

The existence and uniqueness result Theorem 3.1 and the comparison result Theorem 3.5 are basic tools in the forthcoming paper (Geiss and Steinicke 2018) on Malliavin differentiability and boundedness of solutions to BSDEs. To compute the Malliavin derivative for the jump part of the Lévy process, more structure from the generator is required in its dependency on *u*, usually via an integral w.r.t. *ν*(*dx*), for example,

$$ f(s,u) = h \left(s, \int_{\mathbb{R}\setminus\{0\}} u(x) \kappa(s,x) \nu(dx) \right), $$

where $[0,T] \times \mathbb {R} \ni (s,v) \mapsto h(s,v).$ One can find *h* and *κ* such that the assumptions of Theorem 3.5 are satisfied while conditon (2) does not hold: By the mean value theorem there exists a *ζ*∈]0,1[ and

$$v_{\zeta}:= {\int}_{\mathbb{R}\setminus\{0\}} \left(\zeta u(x) + (1-\zeta) u^{\prime}(x)\right) \kappa(s,x) \nu(dx), $$

such that

$$\begin{array}{@{}rcl@{}} f(s, u) - f\left(s, u^{\prime}\right) &=& \partial_{v} h \left(s, v_{\zeta} \right) {\int}_{\mathbb{R}\setminus\{0\}} \left(u(x) - u^{\prime}(x) \right) \kappa(s,x) \nu(dx). \end{array} $$

Assumption (3) holds if $ \gamma _{s}^{u,u{\prime }}(x):= \partial _{v} h \left (s,v_{\zeta }\right) \kappa (s,x) \ge -1$ for all (*s*,*u*,*u*^′^,*x*). Choosing, for example, a bounded function *h* such that also sup*s*,*v*|*∂*_*v*_*h*(*s*,*v*)|<*∞*, but *∂*_*v*_*h*(*s*,*v*)≠0 for a.e. *s* and *v*, and putting $\kappa (s,x) = s^{-\frac {1}{4}} (|x|\wedge 1),$ then (2) does not hold since

$$\sup_{s, u,u^{\prime}} \left|\gamma_{s}^{u,u{\prime}} \right| \notin L^{2}(\nu). $$

However, the Assumptions (A2), (A3) of Section 3 are satisfied for

$$K_{2}(s) = \beta(s) = \sup_{v} \left| \partial_{v} h (s,v)\right| \left\|\kappa(s,\cdot)\right\|_{L^{2}(\nu)} \le c s^{-\frac{1}{4}}. $$

The paper is structured as follows: Section 2 contains preliminaries and basic definitions. In Section 3, we present the main theorems of this paper about existence and uniqueness of solutions, the approximation using BSDEs based on Lévy processes with finite Lévy measure, and the comparison result. The latter we also prove there. Having stated and proved some auxiliary results in Section 4, including an a-priori estimate for our type of BSDEs, we are able to prove existence and uniqueness and the approximation result from Section 3. In the appendix, we recall the Bihari–LaSalle inequality and the Jankov–von Neumann theorem.

## Setting

Let *X*=(*X*_*t*_)_*t*∈[0,*T*]_ be a càdlàg Lévy process on a complete probability space $(\Omega,\mathcal {F},\mathbb {P})$ with Lévy measure *ν*. We will denote the augmented natural filtration of *X* by $\left ({\mathcal {F}_{t}}\right)_{t\in {[0,T]}}$ and assume that $\mathcal {F}=\mathcal {F}_{T}.$ For 0<*p*≤*∞* we use the notation $\left (L^{p},\|\cdot \|_{p}\right):=\left (L^{p}(\Omega,\mathcal {F},\mathbb {P}),\|\cdot \|_{L^{p}}\right)$. Equations or inequalities for objects of these spaces throughout the paper are considered up to $\mathbb {P}$-null sets.

The Lévy–Itô decomposition of a Lévy process *X* can be written as

4 $$ X_{t} = a t + \sigma W_{t} + {\int}_{{]0,t]}\times \{ |x|\le1\}} x\tilde{N}(ds,dx) + {\int}_{{]0,t]}\times \{ |x|> 1\}} x N(ds,dx),  $$

where $a\in \mathbb {R}$, *σ*≥0, *W* is a Brownian motion and *N* ($\tilde N$) is the (compensated) Poisson random measure corresponding to *X*, see Applebaum (2004) or Sato (1999).

**Notation**

- Let $\mathcal {S}^{2}$ denote the space of all $(\mathcal {F}_{t})$-progressively measurable and càdlàg processes $Y\colon \Omega \times {[0,T]} \rightarrow \mathbb {R}$ such that
  $$\begin{array}{@{}rcl@{}} \left\|Y\right\|^{2}_{\mathcal{S}^{2}}:=\mathbb{E}\sup_{0\leq t\leq T} \left|Y_{t}\right|^{2} <\infty. \end{array} $$
- We define *L*^2^(*W*) as the space of all $(\mathcal {F}_{t})$-progressively measurable processes $Z\colon \Omega \times {[0,T]}\rightarrow \mathbb {R}$ such that
  $$\begin{array}{@{}rcl@{}} \left\|Z\right\|_{L^{2}(W) }^2:=\mathbb{E}{\int}_{0}^{T}\left|Z_{s}\right|^{2} ds<\infty. \end{array} $$
- Let $\mathbb {R}_{0}:= \mathbb {R}\!\setminus \!\{0\}$. We define $L^{2}\left (\tilde N\right)$ as the space of all random fields $U\colon \Omega \times {[0,T]}\times {\mathbb {R}_{0}}\rightarrow \mathbb {R}$ which are measurable with respect to $\mathcal {P}\otimes \mathcal {B}\left (\mathbb {R}_{0}\right)$ (where $\mathcal {P}$ denotes the predictable *σ*-algebra on *Ω*×[0,*T*] generated by the left-continuous $(\mathcal {F}_{t})$-adapted processes) such that
  $$\begin{array}{@{}rcl@{}} \left\|U\right\|_{L^{2}\left(\tilde N\right)}^{2}:=\mathbb{E}{\int}_{{[0,T]}\times{\mathbb{R}_{0}}}\left|U_{s}(x)\right|^{2} ds\,\nu(dx)<\infty. \end{array} $$
- $L^{2}(\nu):= L^{2}\left (\mathbb {R}_{0}, \mathcal {B}\left (\mathbb {R}_{0}\right), \nu \right), \|\cdot \|:=\|\cdot \|_{L^{2}(\nu)}.$
- $L^{p}([0,T]):=L^{p}([0,T],\mathcal {B}([0,T]), \lambda)$ for *p*>0, where *λ* is the Lebesgue measure on [0,*T*].
- With a slight abuse of the notation, we define
  5 $$\begin{array}{@{}rcl@{}}  &&  L^{2}\left(\Omega; L^{1}([0,T])\right) \\ & :=&\!\!\!\!\! \left \{F \in L^{0}(\Omega \times [0, T], \mathcal{F} \otimes \mathcal{B}([0,T]), \mathbb{P} \otimes \lambda): \mathbb{E} \!\left[{\int}_{0}^{T} \!\!|F(\omega, t)| dt\right]^{2}\!\! <\! \infty. \right \}  \end{array} $$
  For *F*∈*L*^2^(*Ω*;*L*^1^([0,*T*])), put
  6 $$\begin{array}{@{}rcl@{}}  I_{F}(\omega):= {\int}_{0}^{T} F(\omega, t)dt \quad \text{ and} \quad K_{F}(\omega, s) := \frac{F(\omega, s)}{I_{F}(\omega)}. \end{array} $$
- A **solution to a BSDE** with terminal condition *ξ* and generator *f* is a triplet $(Y,Z,U)\in \mathcal {S}^{2}\times L^{2}(W)\times L^{2}\left (\tilde N\right)$ which satisfies for all *t*∈[0,*T*]:
  7 $$ Y_{t}=\xi+{\int}_{t}^{T} f(s,Y_{s},Z_{s},U_{s})ds-{\int}_{t}^{T} Z_{s} {dW}_{s}-{\int}_{{]t,T]}\times\mathbb{R}_{0}}U_{s}(x)\tilde{N}(ds,dx).  $$
  The BSDE (7) itself will be denoted by (*ξ*,*f*).

## Main results

We start with a result about existence and uniqueness which is proved in Section 5.

### **Theorem 3.1**

There exists a unique solution to the BSDE (*ξ*,*f*) with *ξ*∈*L*^2^ and generator $f:\Omega \times {[0,T]}\times \mathbb {R}\times \mathbb {R}\times L^{2}(\nu)\to \mathbb {R}$ satisfying the properties

1. For all (*y*,*z*,*u*):(*ω*,*s*)↦*f*(*ω*,*s*,*y*,*z*,*u*) is progressively measurable.
2. There are nonnegative, progressively measurable processes *K*_1_,*K*_2_, and *F* with
  8 $$\begin{array}{@{}rcl@{}}  C_{K}:= \left \|{\int}_{0}^{T}\left(K_{1}(\cdot,s)+K_{2}(\cdot,s)^{2}\right)ds \right \|_{\infty}<\infty \end{array} $$
  and *F*∈*L*^2^(*Ω*;*L*^1^([0,*T*])) (see (5)) such that for all (*y*,*z*,*u*),
  $$\begin{array}{@{}rcl@{}} &|f(s,y,z,u)|\leq F(s)+K_{1}(s)|y|+K_{2}(s)(|z|+\|u\|), \quad \mathbb{P}\otimes\lambda\text{-a.e.} \end{array} $$
3. For *λ*-almost all *s*, the mapping (*y*,*z*,*u*)↦*f*(*s*,*y*,*z*,*u*) is $\mathbb {P}$-a.s. continuous. Moreover, there is a nonnegative function *α*∈*L*^1^([0,*T*]), *c*>0 and a progressively measurable process *β* with $\int _{0}^{T} \beta (\omega,s)^{2} ds< c$, $\mathbb {P}$-a.s. such that for all (*y*,*z*,*u*),(*y*^′^,*z*^′^,*u*^′^),
  $$\begin{array}{@{}rcl@{}} &\left(y-y^{\prime}\right)\left(f\left(s,y,z,u\right)-f\left(s,y^{\prime},z^{\prime},u^{\prime}\right)\right)\\ &\leq \alpha(s)\rho\left(|y-y^{\prime}|^{2}\right)+\beta(s)\left|y-y^{\prime}\right|\left(\left|z-z^{\prime}\right|+\left\|u-u^{\prime}\right\|\right), \mathbb{P}\otimes\lambda\text{-a.e.}, \end{array} $$
  where *ρ* is a nondecreasing, continuous and concave function from [0,*∞*[ to itself, satisfying *ρ*(0)=0, and $\int _{0^{+}}\frac {1}{\rho (x)}dx=\infty.$
4. The function *ρ* in (A3) satisfies $\limsup _{x \downarrow 0} \frac {\rho (x^{2})}{x} =0.$

If *f* satisfies only (A1)–(A3), then there exists at most one solution.

For *ρ*(*x*)=*x*, we are in the case of the ordinary monotonicity condition. Another example for a function *ρ* is given by

$$\rho(x)=1-\min\left(x,\tfrac{1}{e}\right)^{\min\left(x,\tfrac{1}{e}\right)},\quad x\geq 0.$$

### **Remark 3.2**

white.

1. Condition *(A2)* implies that *f*(*s*,*y*,*z*,*u*) is integrable for a.e. *s*∈[0,*T*] since, by Fubini’s theorem,
  9 $$\begin{array}{@{}rcl@{}}  {\int}_{0}^T&&  \mathbb{E} |f(s,y,z,u)| ds  \\ &\le& \mathbb{E} {\int}_{0}^{T} [F(s) + K_{1}(s)|y| + K_{2}(s)(|z|+\|u\|))]ds < \infty. \end{array} $$
2. If $\limsup _{x \downarrow 0} \frac {\rho (x^{2})}{x} =0$ is satisfied one can derive Lipschitz continuity of *f*(*s*,*y*,*z*,*u*) in *z* and *u* from the monotonicity condition in *(A3)*. We require *(A4)* since we later want to apply (Yin and Mao (2008), Theorem 2.1), where Lipschitz continuity in *u* is used to show uniqueness of solutions. If only *(A1)–(A3)* are satisfied but not *(A4)*, and a Lipschitz condition in *z*,*u* holds nevertheless, all of the article’s theorems remain valid. One can show that *(A4)* does not follow from the other conditions imposed on *ρ* in *(A3)*: Assume a decreasing sequence $\left (x_{n}\right)_{n=0}^{\infty }$ with *x*_0_=1 and ${\lim }_{n \to \infty } x_{n} =0.$ Define
  $$\rho(x):= \left \{ \begin{array}{ll} \sqrt{x_{n}} &\text{if }\,\,\, x=x_{n}, \, n=0,1,2,... \\ \sqrt{x} &\text{if }\,\,\, x > 1 \text{ or} x=0. \end{array} \right. $$
  and let *ρ* be continuous and piecewise linear on ]0,1]. The so defined *ρ* is a concave function with $\limsup _{x \downarrow 0} \frac {\rho (x)}{\sqrt {x}} =1.$ The sequence $(x_{n})_{n=0}^{\infty }$ can be constructed such that $\int _{0}^{1}\frac {1}{\rho (x)}dx=\infty.$ For example, choose *x*_1_ such that $\int _{x_{1}}^{1}\frac {1}{\rho (x)}dx\ge 1,$ and if *x*_*n*_ has been chosen find *x*_*n*+1_ such that
  $${\int}_{x_{n+1}}^{x_{n}}\frac{1}{\rho(x)}dx = \frac{1}{2}\left(\log(x_{n})-\log(x_{n+1})\right)\left(\sqrt{x_{n}}+\sqrt{x_{n+1}}\right) \ge 1.$$

The next result shows how a solution to a BSDE can be approximated by a sequence of solutions of BSDEs which are driven by Lévy processes with a finite Lévy measure. We do this by approximating the underlying Lévy process defined through

$$X_{t}=at+\sigma W_{t}+{\int}_{]0,t]\times \{|x|>1\}}x N(ds,dx)+{\int}_{]0,t]\times \{|x|\leq 1\}}x \tilde{N}(ds,dx) $$

for *n*≥1 by

$$X^{n}_{t}=at+\sigma W_{t}+{\int}_{]0,t]\times \{|x|>1\}}x N(ds,dx)+{\int}_{]0,t]\times \{1/n \leq |x|\leq 1\}}x \tilde{N}(ds,dx). $$

The process *X*^*n*^ has a finite Lévy measure *ν*_*n*_. Furthermore, note that the compensated Poisson random measure associated with *X*^*n*^ can be expressed as $\tilde {N}^{n}=\chi _{\{1/n \leq |x|\}}\tilde {N}.$ Let

10 $$\begin{array}{@{}rcl@{}}  \mathcal{J}^0&:=&\{\Omega, \emptyset\} \vee \mathcal{N},  \\ \mathcal{J}^n&:=& \sigma\left(X^{n}\right) \vee \mathcal{N}, \quad n\geq 1, \end{array} $$

where $\mathcal {N}$ stands for the null sets of $\mathcal {F}.$ Note that $\left (\mathcal {J}^{n}\right)_{n=0}^{\infty }$ forms a filtration. The notation $\left (\mathcal {J}^{n}\right)_{n=0}^{\infty }$ was chosen to indicate that this filtration describes the inclusion of smaller and smaller *jumps* of the Lévy process. We will use

$$\mathbb{E}_{n} \cdot := \mathbb{E}\left[\ \cdot\ \middle|\mathcal{J}^{n}\right] $$

for the conditional expectation.

The intuitive idea now would be to work with a BSDE driven by *X*^*n*^ where one uses the data $\left (\mathbb {E}_{n} \xi, \mathbb {E}_{n} f\right).$ The problem is that the generator *f* needs to be progressively, and also jointly measurable w.r.t. (*ω*,*t*,*y*,*z*,*u*), but it is not obvious whether the conditional expectation $ \mathbb {E}_{n} f$ preserves this property from *f*. For BSDEs driven by a Brownian motion, this problem has been solved in (Ylinen (2017), Proposition 7.3), but this proposition does not apply to our situtation. Therefore, we next propose a method for the construction of a unique progressively measurable and jointly measurable w.r.t. (*ω*,*t*,*y*,*z*,*u*) version of $ \mathbb {E}_{n} f.$

### **Definition 3.3**

(Definition of *f*_*n*_) Assume that *f* satisfies (A1), (A2) and that $\mathbb {J}:= \left (\mathcal {J}^{[s]}\right)_{s\in [0,\infty [}$ is built using (10), where [·] denotes the floor function. Let $^{o,\mathbb {J}} f$ be the optional projection of the process

$$\begin{aligned} {[0,\infty[}\times\Omega\times{[0,T]}\times\mathbb{R}^{2}\times L^{2}(\nu) \to & \,\mathbb{R},\\ (s,\omega,t,y,z,u)\mapsto& \ f(\omega,t,y,z,u) \end{aligned} $$

in the variables (*s*,*ω*) with respect to $\mathbb {J},$ and with parameters (*t*,*y*,*z*,*u*). For each *n*≥0, assume that the filtration $\mathbb {F}^{n} :=\left (\mathcal {F}_{t}^{n}\right)_{t\in {[0,T]}}$ is given by $\mathcal {F}_{t}^{n}:=\mathcal {F}_{t}\cap \mathcal {J}^{n}.$ Let *f*_*n*_ be the optional projection of

$$(\omega,t,y,z,u)\mapsto {\!~\!}^{\!\!\!o,\mathbb{J}\!\!} f(n,\omega,t,y,z,u)$$

with respect to $\mathbb {F}^{n}$ with parameters (*y*,*z*,*u*).

The reason for using the filtration $\left (\mathcal {J}^{[s]}\right)_{s\in [0,\infty [}$ instead of the $ \left (\mathcal {J}^{n}\right)_{n=0}^{\infty }$ from (10) is that one can apply known measurability results w.r.t. right continuous filtrations instead of proving measurability here directly. Indeed, the optional projection ${\!~\!}^{\!\!\!o,\mathbb {J}\!\!}f$ defined above is jointly measurable in (*s*,*ω*,*t*,*y*,*z*,*u*). For this we refer to Meyer (1979), where optional and predictable projections of random processes depending on parameters were considered, and their uniqueness up to indistinguishability was shown.

It follows that for all (*t*,*y*,*z*,*u*),

$${\!~\!}^{o,\mathbb{J}}f(n,t,y,z,u)=\mathbb{E}_{n} f(t,y,z,u), \quad \mathbb{P}\text{-a.s.} $$

Then, since *f* is $\left (\mathcal {F}_{t}\right)_{t\in {[0,T]}}$-progressively measurable, for all *n*≥0, *t*∈[0,*T*] and all (*y*,*z*,*u*), it holds that

11 $$ f_{n}(t,y,z,u)=\mathbb{E}_{n} f(t,y,z,u), \quad\mathbb{P}\text{-a.s.}  $$

Hence, *f*_*n*_(*t*,*y*,*z*,*u*) is a jointly measurable version of $\mathbb {E}_{n} f(t,y,z,u)$ which is $\left (\mathcal {F}_{t}^{n}\right)_{t\in {[0,T]}}$-optional, so especially it is progressively measurable.

We comment on the compatibility of the solutions (*Y*^*n*^,*Z*^*n*^,*U*^*n*^) from the BSDE corresponding to $\left (\mathbb {E}_{n}\xi,f_{n}\right),$

$$\begin{array}{@{}rcl@{}} Y^{n}_{t} &=&\mathbb{E}_{n} \xi+{\int}_{t}^{T} f_{n}\left(s,Y^{n}_{s},Z^{n}_{s},U^{n}_{s}\right)ds-{\int}_{t}^{T} Z^{n}_{s} {dW}_{s} \\ &&-{\int}_{{]t,T]}\times \mathbb{R}_{0}}U^{n}_{s}(x)\tilde{N}^{n}(ds,dx) \end{array} $$

with the space $S^{2}\times L^{2}(W)\times L^{2}\left (\tilde N\right)$:

The triplet $\left (Y^{n},Z^{n},U^{n}\right)\in S^{2}\times L^{2}(W)\times L^{2}\left (\tilde N^{n}\right)$ can be canonically embedded in the space $S^{2}\times L^{2}(W)\times L^{2}\left (\tilde N\right)$, basically by extending $U^{n}_{s}(x)$ onto $\mathbb {R}_{0}$ by defining $U^{n}_{s}(x):=0$ for $|x|<\frac {1}{n}$. Moreover, recall that $\tilde {N}^{n}=\chi _{\{1/n \leq |x|\}}\tilde {N},$ so that

$${\int}_{{]t,T]}\times \mathbb{R}_{0}}\!\!U^{n}_{s}(x)\tilde{N}^{n}(ds,dx) ={\int}_{{]t,T]}\times \mathbb{R}_{0}}\!\!\!U^{n}_{s}(x) \chi_{\{1/n \leq |x|\}}\tilde{N}(ds,dx). $$

Therefore, $\left (Y^{n},Z^{n},U^{n}\chi _{\mathbb {R}\setminus {]-1/n,1/n[}}\right)$ solves $\left (\mathbb {E}_{n}\xi,f_{n}\right)$ in $S^{2}\times L^{2}(W)\times L^{2}\left (\tilde N\right)$.

### **Theorem 3.4**

Let *ξ*∈*L*^2^ and let *f* satisfy *(A1)*–*(A3)*. Assume that the BSDE driven by *X*^*n*^ with data $\left (\mathbb {E}_{n}\xi,f_{n}\right)$ (where *f*_*n*_ is given by Definition 3.3) has a unique solution denoted by (*Y*^*n*^,*Z*^*n*^,*U*^*n*^). If the solution (*Y*,*Z*,*U*) to (*ξ*,*f*) exists as well, then,

$$\left(Y^{n},Z^{n},U^{n}\right)\to(Y,Z,U) $$

in $L^{2}(W)\times L^{2}(W)\times L^{2}\left (\tilde {N}\right)$ on $(\Omega,\mathcal {F},\mathbb {P})$. Moreover, if *f* additionally satisfies *(A4)*, then the mentioned solution triplets exist.

The benefit of this approximation becomes clear in the proof of the comparison theorem which we state next. There, we only need to prove the comparison result assuming a finite Lévy measure, since the general case then follows by approximation.

Another consequence of this approximation result concerns nonlinear expectations. (For a survey article on nonlinear expectations the reader is referred to Peng (2010)). In the case of Lévy processes, provided that *f*(*s*,*y*,0,0)=0 for all *s* and *y*, the process *Y*_*t*_ has been described by Royer (2006) as a conditional nonlinear expectation, denoted by $\mathbb {E}^{f}_{t} \xi :=Y_{t}.$ Hence, our theorem implies that

$$\left(\mathbb{E}^{f_{n}}_{t} \mathbb{E}_{n}\xi \right)_{t \in [0,T]} \to \left(\mathbb{E}^{f}_{t} \xi \right)_{t \in [0,T]} \quad \text{ in} L^{2}(W). $$

### **Theorem 3.5**

Let *f*,*f*^′^ be two generators satisfying the conditions *(A1)*–*(A3)* of Theorem 3.1 (*f* and *f*^′^ may have different coefficients). We assume *ξ*≤*ξ*^′^, $\mathbb {P}$-a.s. and for all (*y*,*z*,*u*), *f*(*s*,*y*,*z*,*u*)≤*f*^′^(*s*,*y*,*z*,*u*), for $\mathbb {P}\otimes \lambda $-a.a. (*ω*,*s*)∈*Ω*×[0,*T*]. Moreover, assume that *f* or *f*^′^ satisfy the condition (here formulated for *f*)

- (A *γ*) $f(s,y,z,u)- f(s,y,z,u^{\prime })\leq \int _{\mathbb {R}_{0}}\left (u^{\prime }(x)-u(x)\right)\nu (dx), \quad \mathbb {P}\otimes \lambda $-a.e.
  for all *u*,*u*^′^∈*L*^2^(*ν*) with *u*≤*u*^′^.

Let (*Y*,*Z*,*U*) and (*Y*^′^,*Z*^′^,*U*^′^) be the solutions to (*ξ*,*f*) and (*ξ*^′^,*f*^′^), respectively.

Then, $Y_{t}\leq Y^{\prime }_{t}$, $\mathbb {P}$-a.s.

### *Proof*

The basic idea for this proof was inspired by the one of Theorem 8.3 in El Karoui et al. (2009).

Step 1:

In this step we assume that the Lévy measure *ν* is finite. We use Tanaka–Meyer’s formula (cf. Protter (2004), Theorem 70) to see that for $\eta (s):=2\beta (s)^{2}+\nu (\mathbb {R}_{0})$,

$${\begin{aligned} e^{\int_{0}^{t} \eta(s)ds}\left(Y_{t}-Y^{\prime}_{t}\right)^{2}_{+}&=e^{\int_{0}^{T} \eta(s)ds}\left(\xi-\xi^{\prime}\right)^{2}_{+}+M(t)\\ &\quad +{\int}_{t}^{T} e^{\int_{0}^{s} \eta(\tau)d\tau}\chi_{\{Y_{s}-Y^{\prime}_{s}\geq 0\}}\left[2\left(Y_{s}-Y^{\prime}_{s}\right)_{+}\left(f\left(s,Y_{s},Z_{s},U_{s}\right)\right.\right.\\ &\quad\left.-f^{\prime}\left(s,Y^{\prime}_{s},Z^{\prime}_{s}, U^{\prime}_{s}\right)\right)\\ &\quad -\left|Z_{s}-Z^{\prime}_{s}\right|^{2}- \eta(s)\left|Y_{s}-Y^{\prime}_{s}\right|^{2}\\ &\quad-{\int}_{\mathbb{R}_{0}}\left(\left(Y_{s}-Y^{\prime}_{s}+U_{s}(x)-U^{\prime}_{s}(x)\right)^{2}_{+}-\left(Y_{s}-Y^{\prime}_{s}\right)^{2}_{+}\right.\\ &\quad \left.\left.-2\left(U_{s}(x)-U^{\prime}_{s}(x)\right)\left(Y_{s}-Y^{\prime}_{s}\right)_{+}\right)\nu(dx)\right]ds. \end{aligned}} $$

Here, *M*(*t*) is a stochastic integral term having zero expectation which follows from $Y,Y^{\prime }\in \mathcal {S}^{2}$ (this holds according to Theorem 3.1). Moreover, we used that on the set {*ΔY*_*s*_≥0} (where *ΔY*:=*Y*−*Y*^′^) we have $\left (Y_{s}-Y^{\prime }_{s}\right)_{+}=\left |Y_{s}-Y^{\prime }_{s}\right |$. Taking means and denoting the differences by *Δξ*:=*ξ*−*ξ*^′^, *ΔZ*:=*Z*−*Z*^′^, *ΔU*:=*U*−*U*^′^ and *Δf*:=*f*−*f*^′^ leads us to

12 $$ {\begin{aligned} \mathbb{E} e^{\int_{0}^{t} \eta(s)ds}\left(\Delta Y_{t}\right)^{2}_{+}&=\mathbb{E} e^{\int_{0}^{T} \eta(s)ds}(\Delta \xi)^{2}_{+} \\ &\quad+\mathbb{E} \left\{ {\int}_{t}^{T} e^{\int_{0}^{s} \eta(\tau)d\tau}\chi_{\{\Delta Y_{s}\geq 0\}}\left[2\left(\Delta Y_{s}\right)_{+} \left(f\left(s,Y_{s},Z_{s},U_{s}\right)\right.\right.\right. \\ &\quad \left.-f^{\prime}\left(s,Y^{\prime}_{s},Z^{\prime}_{s}, U^{\prime}_{s}\right)\right) -\left|\Delta Z_{s}\right|^{2}- \eta(s)\left|\Delta Y_{s}\right|^{2}\\ &\quad-{\int}_{\mathbb{R}_{0}}\left(\left(\Delta Y_{s}+ \Delta U_{s}(x)\right)^{2}_{+}-\left(\Delta Y_{s}\right)^{2}_{+}\right.\\ &\quad-2\left.\left.\left.\left(\Delta U_{s}(x)\right)\left(\Delta Y_{s}\right)_{+}\right)\nu(dx)\right]ds {\vphantom{{\int}_{t}^{T}}}\right\}, \end{aligned}}  $$

We split up the set $\mathbb {R}_{0}$ into

$$B(\omega,s)=B=\left\{\Delta U_{s}(x)\geq -\Delta Y_{s}\right\}\text{ and} B^{c}. $$

Taking into account that *ξ*≤*ξ*^′^, we estimate

13 $$ {\begin{aligned} \mathbb{E} e^{{\int}_{0}^{t} \eta(s)ds}\left(\Delta Y_{t}\right)^{2}_{+} &\leq \mathbb{E} \left\{ {\int}_{t}^{T} e^{{\int}_{0}^{s} \eta(\tau)d\tau}\chi_{\{\Delta Y_{s}\geq 0\}}\left[{\vphantom{{\int}_{B^{c}}}}2\left(\Delta Y_{s}\right)_{+}\left(f\left(s,Y_{s},Z_{s},U_{s}\right)\right.\right.\right.\\ &\left.\,-\,f^{\prime}\left(s,Y^{\prime}_{s},Z^{\prime}_{s}, U^{\prime}_{s}\right)\right)\,-\,\left|\Delta Z_{s}\right|^{2}\,-\,\eta(s)\left|\Delta Y_{s}\right|^{2}\! -\!{\int}_{B}\!\left|\Delta U_{s}(x)\right|^{2}\nu(dx)\\ &+{\int}_{B^{c}}\!\left.\left.\left((\Delta Y_{s})^{2}_{+}+2(\Delta U_{s}(x))(\Delta Y_{s})_{+}\right)\nu(dx){\vphantom{{\int}_{0}^{t}}}\right]ds {\vphantom{{\int}_{0}^{t}}}\right\}. \end{aligned}}  $$

We focus on the term $(\Delta Y_{s})_{+}\left (f\left (s,Y_{s},Z_{s},U_{s}\right)-f^{\prime }\left (s,Y^{\prime }_{s},Z^{\prime }_{s}, U^{\prime }_{s}\right)\right)$, and denoting ((*Y*,*Z*),(*Y*^′^,*Z*^′^)) by (*Θ*,*Θ*^′^), we derive from *f*≤*f*^′^ that

$${\begin{aligned} (\Delta Y_{s})_{+}\left(f\left(s,\Theta_{s},U_{s}\right)-f^{\prime}\left(s,\Theta^{\prime}_{s}, U^{\prime}_{s}\right)\right) &=(\Delta Y_{s})_{+}\left(f\left(s,\Theta_{s},U_{s}\right)-f\left(s,\Theta^{\prime}_{s}, U^{\prime}_{s}\right)\right.\\ &\quad \left.+f\left(s,\Theta^{\prime}_{s},U^{\prime}_{s}\right)-f^{\prime}\left(s,\Theta^{\prime}_{s}, U^{\prime}_{s}\right)\right)\\ &\quad\!\leq\!(\!\Delta Y_{s}\!)_{+}\left(\!f\left(s,\Theta_{s},U_{s}\right)\,-\,f\left(s,\Theta^{\prime}_{s}, U^{\prime}_{s}\right)\!\right). \end{aligned}} $$

We continue with the observation that on {*ω*:*ΔY*_*s*_>0} we have

$$B^{c} = \left\{ \Delta U_{s}(x)< - \Delta Y_{s}\right\} \subseteq \left\{ U^{\prime}_{s}(x) > U_{s}(x)\right\}, $$

so that

$$U^{\prime}_{s}\chi_{B}+U_{s}\chi_{B^{c}} \le U^{\prime}_{s}\chi_{B}+U^{\prime}_{s}\chi_{B^{c}} \quad \text{ on} \quad \left\{\omega: \Delta Y_{s} >0 \right\}. $$

Therefore, we split $(\Delta Y_{s})_{+}\left (f\left (s,\Theta _{s},U_{s}\right)-f\left (s,\Theta ^{\prime }_{s}, U^{\prime }_{s}\right)\right)$ into two terms; one we estimate with (A3) and the first inequality of (14), while for the other we use (A *γ*):

$${\begin{aligned} (\Delta Y_{s})_{+}\left(f\left(s,\Theta_{s},U_{s}\right)-f\left(s,\Theta^{\prime}_{s}, U^{\prime}_{s}\right)\right) &=(\Delta Y_{s})_{+}\left(f\left(s,\Theta_{s},U_{s}\chi_{B}+U_{s}\chi_{B^{c}}\right)\right.\\ &\quad\left.-f\left(s,\Theta^{\prime}_{s}, U^{\prime}_{s}\chi_{B}+U_{s}\chi_{B^{c}}\right)\right)\\ &\quad+(\Delta Y_{s})_{+}\left(f\left(s,\Theta^{\prime}_{s},U^{\prime}_{s}\chi_{B}+U_{s}\chi_{B^{c}}\right)\right.\\ &\quad \left.-f\left(s,\Theta^{\prime}_{s}, U^{\prime}_{s}\chi_{B}+U^{\prime}_{s}\chi_{B^{c}}\right)\right)\\ &\quad\leq \alpha(s)\rho\left(\left(\Delta Y_{s}\right)_{+}^{2}\right)+\beta(s)^{2}(\Delta Y_{s})_{+}^{2}\\ &\quad+\frac{|\Delta Z_{s}|^{2}}{2}+\frac{\|\Delta U_{s}\chi_{B}\|^{2}}{2}\\ &\quad- {\int}_{\mathbb{R}_{0}}(\Delta Y_{s})_{+} \Delta U_{s}(x)\chi_{B^{c}}\nu(dx). \end{aligned}} $$

Thus, by the last two inequalities, (13) evolves to

$${\begin{aligned} \mathbb{E} e^{{\int}_{0}^{t} \eta(s)ds}(\Delta Y_{t})^{2}_{+} &\leq \mathbb{E} \left\{ {\int}_{t}^{T} e^{{\int}_{0}^{s} \eta (\tau)d\tau}\chi_{\{\Delta Y_{s}\geq 0\}}\left[2\alpha(s){\rho}\left(\left(\Delta Y_{s}\right)_{+}^{2}\right)\,+\,2\beta(s)^{2}(\Delta Y_{s})_{+}^{2}\right.\right.\\ &\quad+|\Delta Z_{s}|^{2} +\left\|\Delta U_{s}\chi_{B}\right\|^{2} -{\int}_{B^{c}}2(\Delta Y_{s})_{+} (\Delta U_{s}(x))\nu(dx)\\ &\quad-|\Delta Z_{s}|^{2}-\eta(s)|\Delta Y_{s}|^{2}-\!{\int}_{B}\!\left|\Delta U_{s}(x)\right|^{2}\nu(dx)\\ &\quad+{\int}_{B^{c}}\left.\left.\left(\left(\Delta Y_{s}\right)^{2}_{+}+2(\Delta Y_{s})_{+}\left(\Delta U_{s}(x)\right)\right)\nu(dx)\right]ds \right\}. \end{aligned}} $$

Because of $\left \|\Delta U_{s}\chi _{B}\right \|^{2}={\int }_{B}\!|\Delta U_{s}(x)|^{2}\nu (dx)$, we cancel out terms and get

$${\begin{aligned} \mathbb{E} e^{{\int}_{0}^{t} \eta (s)ds}(\Delta Y_{t})^{2}_{+}&\leq\! \mathbb{E} \left\{{\int}_{t}^{T} e^{{\int}_{0}^{s} \eta (\tau)d\tau}\chi_{\{\Delta Y_{s}\geq 0\}}\left[\!2\alpha(s){\rho}\left((\Delta Y_{s})_{+}^{2}\right)+2\beta(s)^{2}(\Delta Y_{s})_{+}^{2}\right.\right.\\ &\quad\left.\left. - \eta(s)|\Delta Y_{s}|^{2} +{\int}_{B^{c}}\!(\Delta Y_{s})^{2}_{+}\nu(dx)\right]ds \right\}. \end{aligned}} $$

Bounding ${\int }_{B^{c}}\!(\Delta Y_{s})^{2}_{+}\nu (dx)$ by $\nu (\mathbb {R}_{0})(\Delta Y_{s})^{2}_{+}$, leads us to

$${\begin{aligned} \mathbb{E} e^{{\int}_{0}^{t} \eta (s)ds}(\Delta Y_{t})^{2}_{+} &\leq \mathbb{E} \left\{{\int}_{t}^{T} e^{{\int}_{0}^{s} \eta(\tau)d\tau}\chi_{\{\Delta Y_{s}\geq 0\}}\left[2\alpha(s){\rho}((\Delta Y_{s})_{+}^{2})\right.\right.\\ &\quad\left.\left. +(2\beta(s)^{2}+\nu(\mathbb{R}_{0}))(\Delta Y_{s})_{+}^{2} -\eta(s)|\Delta Y_{s}|^{2}\right]ds \right\}. \end{aligned}} $$

It remains, also using the definition of *η*,

$$\begin{array}{@{}rcl@{}} &\mathbb{E} e^{{\int}_{0}^{t} \eta (s)ds}(\Delta Y_{t})^{2}_{+}\leq\mathbb{E}{\int}_{t}^{T} e^{{\int}_{0}^{s} \eta (\tau)d\tau}2\alpha(s){\rho}\left((\Delta Y_{s})_{+}^{2}\right)ds. \end{array} $$

The term $e^{\int _{0}^{T} \eta (\tau)d\tau }$ is $\mathbb {P}$-a.s. bounded by a constant *C*>0. Thus, by the concavity of *ρ*, we arrive at

$$\begin{array}{@{}rcl@{}} &\mathbb{E}(\Delta Y_{t})^{2}_{+}\leq\mathbb{E} \left[e^{\int_{0}^{t} \eta (s)ds}(\Delta Y_{t})^{2}_{+}\right]\leq\int_{t}^{T} 2C\alpha(s){\rho}\left(\mathbb{E}(\Delta Y_{s})_{+}^{2}\right)ds. \end{array} $$

Then, the Bihari–LaSalle inequality (Proposition 5.2)—a generalization of Gronwall’s inequality—shows that $\mathbb {E}(\Delta Y_{t})^{2}_{+}=0$ for all *t*∈[0,*T*], which is the desired result for $\nu (\mathbb {R}_{0})<\infty $.

Step 2:

The goal of this step is to extend the result of the first step to general Lévy measures. We adapt the notation of Theorem 3.4 for *Y*^*n*^,*Y*^*n*^^′^,*f*_*n*_, and $f_{n}^{\prime }$. Now, we claim that for solutions *Y*^*n*^ and *Y*^*n*^^′^ of $(\mathbb {E}_{n}\xi,f_{n})$ and $\left (\mathbb {E}_{n}\xi ^{\prime },f_{n}^{\prime }\right),$ Step 1 granted that *Y*^*n*^≤*Y*^*n*^^′^: Indeed, *f*_*n*_≤*fn*′ holds by the monotonicity of $\mathbb {E}_{n}$, and also (A *γ*) holds for *f*_*n*_ if it did for *f*. One notes that the process *X*^*n*^ which is related to $(\mathbb {E}_{n}\xi,f_{n})$ and $(\mathbb {E}_{n}\xi ^{\prime },f_{n}^{\prime })$ has a finite Lévy measure *ν*_*n*_ satisfying $\nu _{n}(|x|<\frac {1}{n})=0,$ while in (A *γ*) we still have *ν*. However, the solution processes *U*^*n*^ and *U*^*n*^^′^ are zero for $|x|<\frac {1}{n}$ (see the comment before Theorem 3.4).

Hence, we need (A *γ*) only for *u* and *u*^′^ which are zero for $|x|<\frac {1}{n},$ and for those *u* and *u*^′^ we may replace *ν* by *ν*_*n*_ and then apply Step 1. Finally, the convergence of the sequences to the solutions *Y* and *Y*^′^ of (*ξ*,*f*) and (*ξ*^′^,*f*^′^), respectively, in *L*^2^(*W*) shows *Y*≤*Y*^′^, and our theorem is proven. □

## Auxiliary results

We will frequently use the following basic algebraic inequalities (special cases of Young’s inequality) which hold for all *R*>0:

14 $$ ab\leq \frac{a^{2}}{2R}+\frac{Rb^{2}}{2}\quad\quad\text{and}\quad\quad ab\leq \frac{Ra}{2}+\frac{ab^{2}}{2R}.  $$

The following proposition states, roughly speaking, that for the BSDEs considered here it is sufficient to find solution processes of a BSDE in the (larger) space $L^{2}(W)\times L^{2}(W)\times L^{2}(\tilde {N})$.

### **Proposition 4.1**

If $(Y,Z,U)\in L^{2}(W)\times L^{2}(W)\times L^{2}\left (\tilde {N}\right)$ is a triplet of processes that satisfies the BSDE (*ξ*,*f*) with *ξ*∈*L*^2^ and (A1), (A2), then (*Y*,*Z*,*U*)is a solution to (7), i.e., $(Y,Z,U)\in \mathcal {S}^{2}\times L^{2}(W)\times L^{2}\left (\tilde {N}\right)$. In particular, there exists a constant *C*_1_>0 such that

$$\begin{array}{@{}rcl@{}} &\|Y\|^{2}_{\mathcal{S}^{2}} +\left\|Z\right\|_{L^{2}(W) }^{2} + \left\|U\right\|_{L^{2}\left(\tilde{N}\right)}^{2} \leq e^{C_{1}(1+ C_{K})^{2}} \left(\mathbb{E} |\xi|^{2}+\mathbb{E} I^{2}_{F}\right), \end{array} $$

where *C*_*K*_ was defined in (8) and *I*_*F*_ in (6).

### *Proof*

Since (*Y*,*Z*,*U*) satisfies (7), it holds that

$$\begin{array}{@{}rcl@{}} |Y_{t}|^{2}= &Y_{t}\xi+Y_{t}\int_{t}^{T} f(s,Y_{s},Z_{s},U_{s})ds-Y_{t}\int_{t}^{T} Z_{s} {dW}_{s}\\ &-Y_{t}\int_{{]t,T]}\times\mathbb{R}_{0}}U_{s}(x)\tilde{N}(ds,dx). \end{array} $$

We apply the first inequality of (14), where *Y*_*t*_ takes the role of *a*, to get for an arbitrary *R*>0:

$$\begin{array}{@{}rcl@{}} |Y_{t}|^{2}\leq&\frac{3|Y_{t}|^{2}}{2R}+\frac{R|\xi|^{2}}{2}+\frac{R}{2}\left(\left|\int_{t}^{T} Z_{s} {dW}_{s}\right|^{2}+\left|\int_{{]t,T]}\times\mathbb{R}_{0}}U_{s}(x)\tilde{N}(ds,dx)\right|^{2}\right)\\ &+|Y_{t}|\int_{t}^{T} |f(s,Y_{s},Z_{s},U_{s})|ds. \end{array} $$

Condition (A2) implies

$$\begin{array}{@{}rcl@{}} |Y_{t}|^{2}\leq&\frac{3|Y_{t}|^{2}}{2R}+\frac{R|\xi|^{2}}{2}+\frac{R}{2}\left(\left|\int_{t}^{T} Z_{s} {dW}_{s}\right|^{2}+\left|\int_{{]t,T]}\times\mathbb{R}_{0}}U_{s}(x)\tilde{N}(ds,dx)\right|^{2}\right)\\ &+|Y_{t}|\int_{t}^{T} \left(F(s)+K_{1}(s)|Y_{s}|+K_{2}(s)\left(|Z_{s}|+\|U_{s}\|\right)\right)ds. \end{array} $$

We estimate with the help of the inequalities (14),

$$\begin{array}{@{}rcl@{}} |Y_{t}|F(s) &\leq& K_{F}(s)\left(\frac{|Y_{t}|^{2}}{2R}+\frac{{RI}_{F}^{2}}{2}\right),\\ K_{1}(s)|Y_{t}||Y_{s}|&\leq& K_{1}(s)\left(\frac{|Y_{t}|^{2}}{2R}+\frac{R|Y_{s}|^{2}}{2}\right),\\ |Y_{t}|K_{2}(s)\left(|Z_{s}|+\|U_{s}\|\right)&\leq&\frac{K_{2}(s)^{2}|Y_{t}|^{2}}{2R}+R\left(|Z_{s}|^{2}+\|U_{s}\|^{2}\right). \end{array} $$

Hence,

$${\begin{aligned} |Y_{t}|^{2}&\leq\frac{|Y_{t}|^{2}}{2R}\left(4+\int_{t}^{T}\left(K_{F}(s)+K_{1}(s)+K_{2}(s)^{2}\right)ds\right)+\frac{R|\xi|^{2}}{2}\\ &+\frac{R}{2}\left(\left|\int_{t}^{T} Z_{s} {dW}_{s}\right|^{2}+\left|\int_{{]t,T]}\times\mathbb{R}_{0}}U_{s}(x)\tilde{N}(ds,dx)\right|^{2}\right)\\ &+\frac{R}{2} I_{F}^{2} \int_{t}^{T} K_{F}(s)ds+R\!\int_{t}^{T}\left(|Z_{s}|^{2}+\|U_{s}\|^{2}\right)ds+\!\int_{t}^{T}\!\!\frac{{RK}_{1}(s)|Y_{s}|^{2}}{2}ds. \end{aligned}} $$

Note that $\int _{0}^{T} K_{F}(s)ds =1$ and choose $R=R_{0}:= 5+\int _{0}^{T}\left (K_{1}(s)+K_{2}(s)^{2}\right)ds$ so that

$$\begin{array}{@{}rcl@{}} |Y_{t}|^{2}\leq&R_{0}\left[|\xi|^{2}\,+\, \sup_{t\in{[0,T]}}\!\left(\left|\int_{t}^{T} Z_{s} {dW}_{s}\right|^{2}\!\!+\left|\int_{{]t,T]}\times\mathbb{R}_{0}}U_{s}(x)\tilde{N}(ds,dx)\right|^{2}\right)\right.\\ &\quad \quad \quad \quad\quad \quad \quad \left. +I_{F}^{2}+2\int_{0}^{T}|Z_{s}|^{2}+\|U_{s}\|^{2}ds + \int_{t}^{T} K_{1}(s)|Y_{s}|^{2}ds{\vphantom{\frac{1}{\frac{1}{2}}}} \right]. \end{array} $$

Since *Y* is a càdlàg process, we may apply (46) from the appendix which leads to

$${\begin{aligned} |Y_{t}|^{2}\leq& R_{0} e^{R_{0} \int_{0}^{T} K_{1}(s)ds} \left[|\xi|^{2} +I_{F}^{2}+2\int_{0}^{T}|Z_{s}|^{2}+\|U_{s}\|^{2}ds\right.\\ &+ \sup_{t\in{[0,T]}}\!\left.\left(\left|\int_{t}^{T} Z_{s} {dW}_{s}\right|^{2}\!+\left|\int_{{]t,T]}\times\mathbb{R}_{0}}U_{s}(x)\tilde{N}(ds,dx)\right|^{2}\right) \right]. \end{aligned}} $$

The inequality (*a*+*b*)^2^≤2*a*^2^+2*b*^2^ and then Doob’s martingale inequality used on

$$\begin{array}{@{}rcl@{}} \sup_{t\in{[0,T]}}\left(\left|\!\int_{0}^{T} Z_{s} {dW}_{s}-\!\int_{0}^{t} Z_{s} {dW}_{s}\right|^{2}\right.\\ +\left.\left|\int_{{]0,T]}\times\mathbb{R}_{0}}U_{s}(x)\tilde{N}(ds,dx)-\int_{{]0,t]}\times\mathbb{R}_{0}}U_{s}(x)\tilde{N}(ds,dx)\right|^{2}\right) \end{array} $$

yield, since a.s. *R*_0_≤5+*C*_*K*_ and $\int _{0}^{T} K_{1}(s)ds \le C_{K},$

15 $$\begin{array}{@{}rcl@{}} \mathbb{E}\sup_{t\in{[0,T]}}|Y_{t}|^{2} \leq\ c_{1} \left[\mathbb{E}|\xi|^{2}+ \mathbb{E} I_{F}^{2}+ 12 \mathbb{E}\int_{0}^{T}\left(|Z_{s}|^{2}+\|U_{s}\|^{2}\right)ds\right] \end{array} $$

with

16 $$\begin{array}{@{}rcl@{}}  c_{1}=(5 + C_{K})e^{(5+C_{K})C_{K}}. \end{array} $$

For a progressively measurable process *η*, which we will determine later, Itô’s formula implies that

17 $$\begin{array}{@{}rcl@{}} &{|Y_{0}|}^{2}+\int_{0}^{T} e^{\int_{0}^{s}\eta(\tau)d\tau}\left(\eta(s){|Y_{s}|}^{2} +{|Z_{s}|}^{2} +\|U_{s}\|^{2}\right)ds\\ & =M(0)+e^{\int_{0}^{T}\eta(s)ds}|\xi|^{2}+\int_{0}^{T} 2e^{\int_{0}^{s}\eta(\tau)d\tau}Y_{s}f(s,Y_{s},Z_{s},U_{s})ds, \end{array} $$

where

18 $$ \begin{aligned} M(t)&=-\int_{t}^{T}2e^{\int_{0}^{s}\eta(\tau)d\tau}Y_{s}Z_{s}{dW}_{s}\\ &\quad-\int_{{]t,T]}\times\mathbb{R}_{0}}2e^{\int_{0}^{s}\eta(\tau)d\tau}\left((Y_{s-}+ U_{s}(x))^{2}-Y_{s-}^{2}\right)\tilde{N}(ds,dx). \end{aligned}  $$

Provided that $\left \| \int _{0}^{T}\eta (\tau)d\tau \right \|_{L^{\infty }(\mathbb {P})} < \infty,$ one gets $\mathbb {E} M(t)=0$ as a consequence of (15) and the Burkholder–Davis–Gundy inequality (see, for instance, (He et al. (1992), Theorem 10.36)), where the term $\left ((Y_{s-}+ U_{s}(x))^{2}-Y_{s-}^{2}\right)^{2}$ appearing in the integrand can be estimated by

$$\left(|Y_{s-}+ U_{s}(x)|+|Y_{s-}|\right)^{2} \left(|Y_{s-}+ U_{s}(x)|-|Y_{s-}|\right)^{2} \le 4 \sup_{r \in [0,T]} |Y_{r}|^{2} \,|U_{s}(x)|^{2}. $$

By (A2) and (14), we have

$$\begin{array}{@{}rcl@{}} |Y_{s}| |f(s,Y_{s},Z_{s},U_{s})| &\le & |Y_{s}|[F(s)+K_{1}(s)|Y_{s}|+K_{2}(s)(|Z_{s}|+\|U_{s}\|) ]\\ &\le & F(s) |Y_{s}| + K_{1}(s)|Y_{s}|^{2} +2R \frac{ K_{2}(s)^{2} |Y_{s}|^{2}}{2} \\ &&+ \frac{|Z_{s}|^2+ \|U_{s}\|^{2}}{2 R}. \end{array} $$

We use this estimate for *R*=2, and taking the expectation in (17), we have

19 $$ {\begin{aligned} \mathbb{E}\int_{0}^{T}\! \!e^{\int_{0}^{s}\eta(\tau)d\tau}\!\left(\!\eta(s){|Y_{s}|}^{2}\,+\,{|Z_{s}|}^{2}\! +\!\|U_{s}\|^{2}\!\right)ds & \!\leq \!\mathbb{E} e^{\int_{0}^{T}\eta(s)ds}|\xi|^{2}\,+\,\mathbb{E}\int_{0}^{T} \!\!\! e^{\int_{0}^{s}\eta(\tau)d\tau}\left(\frac{|Z_{s}|^{2} +\|U_{s}\|^{2}}{2} \right)ds \\ &\quad+2\mathbb{E}\int_{0}^{T} e^{\int_{0}^{s}\eta(\tau)d\tau}F(s)ds\sup_{t\in{[0,T]}}|Y_{t}|\\ &\quad +\mathbb{E}\int_{0}^{T} e^{\int_{0}^{s}\eta(\tau)d\tau} 2\left(K_{1}(s)+ 2K_{2}(s)^{2}\right)Y_{s}^{2}ds. \end{aligned}}  $$

Then, we choose *η*(*s*)=2(*K*_1_(*s*)+2*K*_2_(*s*)^2^) and subtract the terms containing *Y*,*Z*, and *U* from the left hand side of (19). Moreover, we apply the first inequality of (14) to the term containing the supremum. It follows that

20 $$ {\begin{aligned} \mathbb{E}\int_{0}^{T} e^{\int_{0}^{s}\eta(\tau)d\tau}\left({|Z_{s}|}^{2} +\|U_{s}\|^{2}\right)ds & \leq 2\mathbb{E} \left[e^{\int_{0}^{T}\eta(s)ds}|\xi|^{2}\right] + 2R\mathbb{E}\left[\int_{0}^{T} e^{\int_{0}^{s}\eta(\tau)d\tau}F(s)ds\right]^{2}\\ &\quad+\frac{2}{R}\mathbb{E}\sup_{t\in{[0,T]}}|Y_{t}|^{2}. \end{aligned}}  $$

Note that

$$\mathbb{E}\int_{0}^{T} \left({|Z_{s}|}^{2} +\|U_{s}\|^{2}\right)ds\leq\mathbb{E}\int_{0}^{T} e^{\int_{0}^{s}\eta(\tau)d\tau}\left({|Z_{s}|}^{2} +\|U_{s}\|^{2}\right)ds. $$

Hence, by (20) and $\int _{0}^{T}\eta (\tau)d\tau \le 4C_{K}$ a.s., we have

21 $$\begin{array}{@{}rcl@{}}  \mathbb{E}\int_{0}^{T} \left({|Z_{s}|}^{2} +\|U_{s}\|^{2}\right)ds \leq 2 e^{4C_K} \mathbb{E} |\xi|^{2}+ 2R e^{8C_K} \mathbb{E} I_{F}^{2}+\frac{2}{R}\mathbb{E}\sup_{t\in{[0,T]}}|Y_{t}|^{2}. \end{array} $$

Now, we can plug in (21) into (15) and vice versa which yields for *R*:=48*c*_1_ that

$$\begin{array}{@{}rcl@{}} \mathbb{E}\sup_{t\in{[0,T]}}|Y_{t}|^{2}\leq&\ \left(2c_{1}+48c_{1}e^{4C_{K}}\right)\mathbb{E}|\xi|^{2}+ \left(2c_{1}+(48c_{1})^{2}e^{8C_{K}}\right) \mathbb{E} I_{F}^{2}, \end{array} $$

and

$$\begin{array}{@{}rcl@{}} \mathbb{E}\!\!\int_{0}^{T} \!\!\!\left({|Z_{s}|}^{2} +\|U_{s}\|^{2}\right)\!ds \leq \!\left(\frac{1}{12} + 4 e^{4C_{K}}\right)\! \mathbb{E} |\xi|^{2}\,+\, \left(\frac{1}{12} + 192 c_{1} e^{8C_{K}} \right) \mathbb{E} I_{F}^{2}. \end{array} $$

Using (16) it is easy to see that there exists a constant *C*_1_>0 such that each factor in front of the expectations on the right side of the previous two inequalities is less than $\phantom {\dot {i}\!}e^{C_{1}(1+C_{K})^{2}}.$ □

Our next proposition will be an *L*^2^ a-priori estimate for BSDEs of our type. For the Brownian case, *L*^*p*^ a-priori estimates are done for *p*∈[1,*∞*[ in Briand et al. (2003), and for quadratic BSDEs, for *p*∈[2,*∞*[ in Geiss and Ylinen (2018). For BSDEs with jumps, for *p*∈]1,*∞*[, see Kruse and Popier (2016, 2017); while Becherer et al. (2018) contains an a-priori estimate w.r.t. *L*^*∞*^. The following assertion is similar to (Barles et al. (1997), Proposition 2.2), but fits our extended setting.

### **Proposition 4.2**

Let *ξ*,*ξ*^′^∈*L*^2^ and let *f*,*f*^′^ be two generator functions satisfying (A1)–(A3), where the bounds in (A2) and the coefficients in (A3) may differ for *f* and *f*^′^. The coefficients of *f*^′^ in (A3) will be referred to as *α*^′^ and *β*^′^. Moreover, let the triplets (*Y*,*Z*,*U*) and $(Y^{\prime },Z^{\prime },U^{\prime })\in L^{2}(W)\times L^{2}(W)\times L^{2}\left (\tilde {N}\right)$, satisfy the BSDEs (*ξ*,*f*) and (*ξ*^′^,*f*^′^), respectively.

Then,

$$\begin{array}{@{}rcl@{}} &\|Y-Y^{\prime}\|^{2}_{L^{2}(W)} +\left\|Z-Z^{\prime}\right\|_{L^{2}(W) }^{2} + \left\|U-U^{\prime}\right\|_{L^{2}(\tilde N) }^{2} \\ &\leq h\left(a,b,\mathbb{E}|\xi-\xi^{\prime}|^{2}+2\mathbb{E}\!\int_{0}^{T}\!|Y_{t}-Y^{\prime}_{t}|\left|f(t,Y_{t},Z_{t},U_{t})-f^{\prime}(t,Y_{t},Z_{t},U_{t})\right|dt\right)\!, \end{array} $$

where $a=\int _{0}^{T}\alpha ^{\prime }(s)ds, b=\left \|\int _{0}^{T}\beta ^{\prime }(s)^{2}ds\right \|_{\infty },$ and

$$h:]0,\infty[\times]0,\infty[\times [0,\infty[\to [0,\infty[ $$

is a function such that *h*(*a*,*b*,*x*)→0=*h*(*a*,*b*,0) if *x*→0.

### *Proof*

We start with the following observation gained by Itô’s formula for the difference of the BSDEs (*ξ*,*f*) and (*ξ*^′^,*f*^′^). We denote differences of expressions by *Δ*. If *η*=4*β*^′^(*s*)^2^, we have analogously to (17)

22 $$\begin{array}{@{}rcl@{}}  & e^{\int_{0}^{t}\eta(s)ds}|\Delta Y_{t}|^{2}+\int_{t}^{T} e^{\int_{0}^{s}\eta(\tau)d\tau}\left(\eta(s)|\Delta Y_{s}|^{2} +|\Delta Z_{s}|^{2}+ \|\Delta U_{s}\|^{2}\right)ds \\ &=e^{\int_{0}^{T}\eta(s)ds}|\Delta\xi|^{2}+M(t)  \\ & \quad+\int_{t}^{T} 2e^{\int_{0}^{s}\eta(\tau)d\tau}\Delta Y_{s} \,(f(s,Y_{s},Z_{s},U_{s})-f^{\prime}(s,Y^{\prime}_{s},Z^{\prime}_{s},U^{\prime}_{s}))ds, \end{array} $$

where

$$\begin{aligned} M(t) &= -\int_{t}^{T} 2e^{\int_{0}^{s}\eta(\tau)d\tau}\Delta Y_{s}\Delta Z_{s} {dW}_{s} \\ &\quad-\int_{{]t,T]}\times\mathbb{R}_{0}}2e^{\int_{0}^{s}\eta(\tau)d\tau} \left((\Delta Y_{s-}+\Delta U_{s}(x))^{2}-\Delta Y_{s-}^{2}\right)\tilde{N}(ds,dx). \end{aligned} $$

By the same reasoning as for (18), we have $\mathbb {E}M(t)=0$. We now proceed with the (standard) arguments similar to those used for (17)–(19). By (A3) and the first inequality from (14),

23 $$ {\begin{aligned} \Delta Y_{s} (f^{\prime}(s,Y_{s},Z_{s},U_{s})&-f^{\prime}(s,Y^{\prime}_{s},Z^{\prime}_{s}, U^{\prime}_{s})) \le \alpha^{\prime}(s)\rho\left(|\Delta Y_{s}|^{2}\right)+\beta^{\prime}(s)|\Delta Y_{s}|(|\Delta Z_{s}|\\ &+\|\Delta U_{s}\|) \le\! \alpha^{\prime}(s)\rho\left(|\Delta Y_{s}|^{2}\right)\\ &+ \frac{ \beta^{\prime}(s)^{2}|\Delta Y_{s}|^{2}}{R}\! +\!\frac{R \left(|\Delta Z_{s}|^{2}+\|\Delta U_{s}\|^{2}\right)}{2}. \end{aligned}}  $$

Taking the expectation in (22) and then using (23) with *R*=1 (such that we can cancel out the terms with *Z* and *U* on the left side), leads to

$${\begin{aligned} \mathbb{E} e^{\int_{0}^{t}\eta(s)ds}|\Delta Y_{t}|^{2}&+\mathbb{E} \int_{t}^{T} e^{\int_{0}^{s}\eta(\tau)d\tau}\eta(s)|\Delta Y_{s}|^{2}ds \leq\mathbb{E} e^{\int_{0}^{T}\eta(s)ds}|\Delta\xi|^{2}\\ & +\mathbb{E}\int_{t}^{T} 2 e^{\int_{0}^{s}\eta(\tau)d\tau}\Delta Y_{s} \cdot(\Delta f)(s,Y_{s},Z_{s},U_{s})ds\\ & +\mathbb{E}\int_{t}^{T} e^{\int_{0}^{s}\eta(\tau)d\tau}\left(2\alpha^{\prime}(s)\rho\left(|\Delta Y_{s}|^{2}\right)+ \beta^{\prime}(s)^{2}|\Delta Y_{s}|^{2}\right)ds. \end{aligned}} $$

The choice *η*(*s*)=4*β*^′^(*s*)^2^ and the fact that $ \int _{0}^{T}\beta ^{\prime }(s)^{2}ds \le b$ a.s. leads to

$${\begin{aligned} E|\Delta Y_{t}|^{2}&\leq e^{4b}\left(\mathbb{E} |\Delta\xi|^{2} +\mathbb{E}\int_{t}^{T} 2|\Delta Y_{s}| |(\Delta f)(s,Y_{s},Z_{s},U_{s})|ds\right)\\ &+e^{4b}\int_{t}^{T} 2\alpha^{\prime}(s)\rho\left(\mathbb{E}|\Delta Y_{s}|^{2}\right)ds, \end{aligned}} $$

since *ρ* is a concave function.

By Proposition 5.2, a backward version of the Bihari–LaSalle inequality, shows

24 $$ {\begin{aligned} &\sup_{t\in{[0,T]}}\mathbb{E}|\Delta Y_{t}|^{2}\leq \\ &G^{-1}\!\left\{G\!\left[\!e^{4b}\left(\mathbb{E}|\Delta\xi|^{2}\,+\,\mathbb{E}\int_{0}^{T} \!\!2|\Delta Y_{s}| |(\Delta f)(s,Y_{s},Z_{s},U_{s})|ds\right)\!\right]\,+\,2e^{4b}\!\int_{0}^{T}\!\! \alpha^{\prime}(s)ds\right\}, \end{aligned}}  $$

where $G(x)=\int _{1}^{x}\frac {1}{{\rho }(h)}dh.$

If we take the expectation in (22) but choose this time (23) with $R=\frac {1}{2}$ and omit $\mathbb {E} e^{\int _{0}^{t}\eta (s)ds}|\Delta Y_{t}|^{2},$ then

$${\begin{aligned} & \mathbb{E}\int_{t}^{T} e^{\int_{0}^{s}\eta(\tau)d\tau}\left(\eta(s)|\Delta Y_{s}|^{2}+|\Delta Z_{s}|^{2}+\|\Delta U_{s}\|^{2}\right)ds\\ &\leq \mathbb{E} e^{\int_{0}^{T}\eta(s)ds}|\Delta\xi|^{2} +\mathbb{E}\int_{t}^{T} 2 e^{\int_{0}^{s}\eta(\tau)d\tau}\Delta Y_{s} \cdot(\Delta f)(s,Y_{s},Z_{s},U_{s})ds\\ &+\mathbb{E}\! \left\{\!\int_{t}^{T}\! \!e^{\int_{0}^{s}\eta(\tau)d\tau}\!\left(2\alpha^{\prime}(s)\rho\left(|\Delta Y_{s}|^{2}\right)+ 4 \beta^{\prime}(s)^{2}|\Delta Y_{s}|^{2} +\frac{|\Delta Z_{s}|^{2}+\|\Delta U_{s}\|^{2}}{2}\right)ds \right\}. \end{aligned}} $$

We subtract the quadratic terms with *ΔY*,*ΔZ*, and *ΔU* which appear on the right hand side. This results in the inequality

$$\begin{array}{@{}rcl@{}} & \mathbb{E}\int_{t}^{T} e^{\int_{0}^{s}\eta(\tau)d\tau}\left(|\Delta Z_{s}|^{2}+\|\Delta U_{s}\|^{2}\right)ds\\ &\leq 2\left(\mathbb{E} e^{\int_{0}^{T}\eta(s)ds}|\Delta\xi|^{2} +\mathbb{E}\int_{t}^{T} 2 e^{\int_{0}^{s}\eta(\tau)d\tau}|\Delta Y_{s}| \cdot|(\Delta f)(s,Y_{s},Z_{s},U_{s})|ds\right.\\ &\quad\quad\left.+\mathbb{E}\int_{t}^{T}\! e^{\int_{0}^{s}\eta(\tau)d\tau}2\alpha^{\prime}(s)\rho\left(|\Delta Y_{s}|^{2}\right))ds\right). \end{array} $$

We continue our estimate by

25 $$\begin{array}{@{}rcl@{}} &\mathbb{E}\int_{t}^{T} e^{\int_{0}^{s}\eta(\tau)d\tau}\left(|\Delta Z_{s}|^{2}+\|\Delta U_{s}\|^{2}\right)ds \\ &\leq 2e^{4b}\left(\mathbb{E}|\Delta\xi|^{2} +\mathbb{E}\int_{t}^{T} 2|\Delta Y_{s}| \cdot|(\Delta f)(s,Y_{s},Z_{s},U_{s})|ds\right.\\ &\quad +2\int_{t}^{T} \alpha^{\prime}(s)ds \, {\rho}\left.\left(\underset{s\in{[0,T]}}{\sup}\mathbb{E}|\Delta Y_{s}|^{2}\right)\right), \end{array} $$

since *η*(*s*)=4*β*^′^(*s*)^2^. We put

$$\begin{array}{@{}rcl@{}} H:=& G^{-1}\left\{G\left[e^{4b}\left(\mathbb{E}|\Delta\xi|^{2} +\mathbb{E}\int_{0}^{T} 2|\Delta Y_{s}| |(\Delta f)(s,Y_{s},Z_{s},U_{s})|ds\right)\right]\right. \\ &+\left.2e^{4b}\int_{0}^{T} \alpha^{\prime}(s)ds\right\} \end{array} $$

so that (24) reads now as $ \sup _{t\in {[0,T]}}\mathbb {E}|\Delta Y_{t}|^{2} \leq H.$ If we add this inequality to (25) and note that ${\rho }\left (\sup _{t\in {[0,T]}}\mathbb {E}|\Delta Y_{s}|^{2}\right)\le \rho (H),$ we have

$$\begin{array}{@{}rcl@{}} & \underset{s\in{[0,T]}}{\sup}\mathbb{E}|\Delta Y_{t}|^{2}+\mathbb{E}\int_{0}^{T} |\Delta Z_{s}|^{2}ds+\mathbb{E}\int_{0}^{T} \|\Delta U_{s}\|^{2}ds \\ \leq& 2e^{4b}\left(\mathbb{E}|\Delta\xi|^{2}+\mathbb{E}\int_{0}^{T} 2|\Delta Y_{s}| \cdot|(\Delta f)(s,Y_{s},Z_{s},U_{s})|ds\right)\\ &+\left(2e^{4b}\int_{0}^{T}\alpha^{\prime}(s)ds+1\right)\cdot (\text{id}+{\rho})(H). \end{array} $$

Note that the integral condition on *ρ* implies that, if the argument of *G* approaches zero, then the right hand side vanishes. □

The following Lemma will be used to estimate the expectation of integrals which contain |*Y*_*s*_|^2^.

### **Lemma 4.3**

Let *ξ*∈*L*^2^ and assume that (A1) and (A2) hold. If (*Y*,*Z*,*U*) is a solution to (*ξ*,*f*) and *H* is a nonnegative, progressively measurable process with $\left \|\int _{0}^{T} H(s)ds \right \|_{\infty } < \infty,$ then

26 $$  \begin{aligned} \mathbb{E} \int_{0}^{T} H(s) |Y_{s}|^{2}ds\leq \,& e^{2C_{K}} \mathbb{E}\int_{0}^{T} H(s)ds|\xi|^{2}\\ &+ 2 e^{2C_{K}} \left \|\int_{0}^{T} H(s)ds \cdot I_{F}\right\|_{2}\|Y\|_{\mathcal{S}^{2}}. \end{aligned}  $$

### *Proof*

From the relations (17), (18) and integration by parts applied to the term $\int _{0}^{T} H(s)ds \cdot e^{\int _{0}^{T} \eta (s)ds}|Y_{T}|^{2}, $ we get

$${\begin{aligned} \int_{0}^{T} H(s)ds \cdot e^{\int_{0}^{T} \eta(r)dr}|Y_{T}|^{2} &= \int_{0}^{T} H(s) e^{\int_{0}^{s} \eta(\tau)d\tau}|Y_{s}|^{2} ds - \int_{0}^{T} \left(\int_{0}^{s} H(\tau)d\tau\right) dM(s)\\ &\quad+ \int_{0}^{T} \left(\int_{0}^{s} H(r)dr \right) e^{\int_{0}^{s}\eta(\tau)d\tau} \left(\eta(s){|Y_{s}|}^{2} +{|Z_{s}|}^{2} +\|U_{s}\|^{2} \right.\\ &\quad \left.-2Y_{s}f(s,Y_{s},Z_{s},U_{s}) \right)ds. \end{aligned}} $$

We take expectations and rearrange the equation so that

$${\begin{aligned} \mathbb{E}\!\int_{0}^{T}\! H(s) e^{\int_{0}^{s} \eta(\tau)d\tau} |Y_{s}|^{2}ds &\leq \mathbb{E}\left[\int_{0}^{T} \! H(s)ds\cdot e^{\int_{0}^{T} \eta(s)ds}|\xi|^{2}\right]\\ &+\mathbb{E}\left[\int_{0}^{T}\left(\int_{0}^{s}H(\tau)d\tau\right) e^{\int_{0}^{s} \eta(\tau)d\tau} \!\left(2Y_{s}f(s,Y_{s},Z_{s},U_{s})\right.\right. \\ &\left.\left.-\eta(s)|Y_{s}|^{2}-|Z_{s}|^{2}-\|U_{s}\|^{2}\right)ds{\vphantom{\int_{0}^{T}}}\right]. \end{aligned}} $$

By Assumption (A2) and (14), we have

$${\begin{aligned} 2Y_{s}f(s,Y_{s},Z_{s},U_{s}) &\le 2 |Y_{s}| F(s)+2K_{1}(s)|Y_{s}|^{2}\\ &+ 2K_{2}(s) |Y_{s}| (|Z_{s}|+\|U_{s}\|) \le 2 |Y_{s}| F(s)\\ &+2K_{1}(s)|Y_{s}|^2+ 2 K_{2}(s)^{2} |Y_{s}|^{2} + |Z_{s}|^2+\|U_{s}\|^{2}, \end{aligned}} $$

so that for *η*(*s*)=2*K*_1_(*s*)+2*K*_2_(*s*)^2^ it follows

27 $$ \begin{aligned} \mathbb{E}\!\int_{0}^{T}\! H(s) |Y_{s}|^{2}ds &\leq \mathbb{E}\left[\int_{0}^{T} H(s)ds\cdot e^{\int_{0}^{T} \eta(s)ds}|\xi|^{2}\right]\\ &+ 2\mathbb{E}\left[\int_{0}^{T}\left(\int_{0}^{s}H(\tau)d\tau\right) e^{\int_{0}^{s} \eta(\tau)d\tau}F(s)|Y_{s}|ds\right] \\ & \leq e^{2C_{K}} \mathbb{E}\left[\int_{0}^{T} H(s)ds \cdot |\xi|^{2}\right]\\ &+ 2 e^{2C_{K}} \left \|\int_{0}^{T} H(s)ds\cdot I_{F}\right\|_{2}\|Y\|_{\mathcal{S}^{2}}. \end{aligned}  $$

□

## Proofs of Theorems 3.1 and 3.4

### Proof of Theorem 3.1

Step 1: Uniqueness

Uniqueness of the solution is a consequence of Proposition 4.2, since the terms |*ξ*−*ξ*^′^| and |*f*(*s*,*Y*_*s*_,*Z*_*s*_,*U*_*s*_)−*f*^′^(*s*,*Y*_*s*_,*Z*_*s*_,*U*_*s*_)| are zero.

The proof of existence will be split up in further steps.

Step 2:

In this step, we construct an approximating sequence of generators *f*^(*n*)^ for *f* and show several estimates for the solution processes (*Y*^*n*^,*Z*^*n*^,*U*^*n*^) to the BSDEs (*ξ*,*f*^(*n*)^).

For *n*≥1, define *c*_*n*_(*z*):= min(max(−*n*,*z*),*n*) and $\tilde {c}_{n}(u)\in L^{2}(\nu)$ to be the projection of *u* onto {*v*∈*L*^2^(*ν*):∥*v*∥≤*n*}. Let (*Y*^*n*^,*Z*^*n*^,*U*^*n*^) be the unique solution of the BSDE (*ξ*,*f*^(*n*)^), with the definitions

$$\hat{f}^{(n)}(\omega,s,y,z,u):=f(\omega,s,y,c_{n}(z),\tilde c_{n}(u)),$$

and

$$\begin{array}{@{}rcl@{}} &f^{(n)}(\omega,s,y,z,u):=\text{sign}\left(\hat{f}^{(n)}(\omega,s,y,z,u)\right)\\ &\times\left [ F(\omega, s)\wedge n +(K_{1}(\omega,s)\wedge n)|y| +(K_{2}(\omega,s)\wedge n)(|c_{n}(z)|+\left\|\tilde{c}_{n}(u)\right\|)\right] \end{array} $$

$$\begin{array}{@{}rcl@{}} \text{ if} \quad |\hat{f}^{(n)}(\omega,s,y,z,u)|>\ &F(\omega, s)\wedge n +(K_{1}(\omega,s)\wedge n)|y|\\ \nopagebreak[4] &+(K_{2}(\omega,s)\wedge n)(|c_{n}(z)|+\|\tilde{c}_{n}(u)\|), \end{array} $$

and

$$f^{(n)}(\omega,s,y,z,u):=\hat{f}^{(n)}(\omega,s,y,z,u) \quad \text{else.} $$

Note that *f*^(*n*)^ satisfies (A1)–(A4), with the same coefficients as *f*. Moreover, by (A4), *f*^(*n*)^ satisfies a Lipschitz condition with respect to *u* (see Remark 3.2). Thus, thanks to (Yin and Mao (2008), Theorem 2.1), (*ξ*,*f*^(*n*)^) has a unique solution (*Y*^*n*^,*Z*^*n*^,*U*^*n*^). Moreover, by Proposition 4.1, we get that

28 $$\begin{array}{@{}rcl@{}}  \|Y^{n}\|^{2}_{\mathcal{S}^{2}} +\left\|Z^{n}\right\|_{L^{2}(W)}^{2} + \left\|U^{n}\right\|_{L^{2}(\tilde N)}^{2} \leq e^{C_{1}(1+ C_{K})^{2}} \left(\mathbb{E}|\xi|^{2}+\mathbb{E} I_{F}^{2}\right)<\infty, \end{array} $$

uniformly in *n*. This implies that the families

$$\left({\sup_{t\in{[0,T]}}|Y_{t}^{n}|},n\geq 0\right), \left({|Y^{n}|},n\geq 0\right)\text{ and }\left(|Z^{n}|+\|U^{n}\|,n\geq 0\right) $$

are uniformly integrable with respect to $\mathbb {P}$, $\mathbb {P}\otimes \lambda $ and $\mathbb {P}\otimes \lambda $, respectively.

Step 3:

The goal of this step is to use Proposition 4.2 to get convergence of (*Y*^*n*^,*Z*^*n*^,*U*^*n*^)_*n*_ in $L^{2}(W)\times L^{2}(W)\times L^{2}(\tilde N)$ for a subsequence *n*_*k*_*↑∞* if $\delta _{n_{k},n_{l}} \to 0$ for *k*>*l*→*∞*, where

$$\begin{array}{@{}rcl@{}} \delta_{n,m}&:=& \mathbb{E}\int_{0}^{T} |Y^{n}_{s}-Y^{m}_{s}||f^{(n)}\left(s,Y^{n}_{s},Z^{n}_{s},U^{n}_{s}\right)-f^{(m)}\left(s,Y^{n}_{s},Z^{n}_{s},U^{n}_{s}\right)|ds.\\ \end{array} $$

We observe that the difference of the generators is zero if two conditions are satisfied at the same time: First, if $|Z^{n}|,\|U^{n}_{s}\|< n$, and additionally, by the cut-off procedure for *F*,*K*_1_,*K*_2_, if

$$n>\max\left({F}(\omega,s),K_{1}(\omega,s), K_{2}(\omega,s)\right)=:k(\omega,s). $$

Thus, putting

29 $$\begin{array}{@{}rcl@{}}  \chi_{n} (s) := \chi_{\{ |Z^{n}_{s}|> n\} \cup \{\|U^{n}_{s}\| >n\} \cup \{k(s)>n \}}, \end{array} $$

we have

$${\begin{aligned} \delta_{n,m} &= \mathbb{E}\int_{0}^{T} |Y^{n}_{s}-Y^{m}_{s}||f^{(n)}\left(s,Y^{n}_{s},Z^{n}_{s},U^{n}_{s}\right)-f^{(m)}\left(s,Y^{n}_{s},Z^{n}_{s},U^{n}_{s}\right)|\chi_{n}(s)ds\\ &\le \mathbb{E} \!\left\{\int_{0}^{T} 2|Y^{n}_{s}\,-\,Y^{m}_{s}|\, \chi_{n}(s) \!\times\!\left({F}(s)+K_{1}(s)|Y^{n}_{s}|\,+\,K_{2}(s)\left(|Z^{n}_{s}|+\|U^{n}_{s}\|\right)\right)ds \right\}\!, \end{aligned}} $$

due to the linear growth condition (A2). We estimate this further by

30 $$ {\begin{aligned} \delta_{n,m} &\le \mathbb{E}\int_{0}^{T}\chi_{n}(s)\, F(s)ds \, \left(\sup_{r\in{[0,T]}}| Y^{n}_{r}|+\sup_{r\in{[0,T]}}|Y^{m}_{r}|\right) \\ &\quad + \mathbb{E}\int_{0}^{T}\chi_{n}(s)\,K_{2}(s)\left(| Y^{n}_{s}|+|Y^{m}_{s}|\right)\left(|Z^{n}_{s}|+\|U^{n}_{s}\|\right)ds \\ &\quad +\mathbb{E}\int_{0}^{T} 2|Y^{n}_{s}-Y^{m}_{s}|\, |Y^{n}_{s}| \chi_{n}(s) \,K_{1}(s)ds \\ &=: \delta^{(1)}_{n,m} + \delta^{(2)}_{n,m}+ \delta^{(3)}_{n,m}. \end{aligned}}  $$

For $\delta ^{(1)}_{n,m},$ we use the Cauchy–Schwarz inequality,

$$\delta^{(1)}_{n,m} \le 2 \left (\mathbb{E} \left |\int_{0}^{T}\chi_{n}(s)\, F(s)ds\right |^{2} \right)^{\frac{1}{2}} \left(\|Y^{n}\|_{\mathcal{S}^{2}}+\|Y^{m}\|_{\mathcal{S}^{2}}\right). $$

Since $\sup _{n} \|Y^{n}\|_{\mathcal {S}^{2}}< \infty $ according to (28), it remains to show that the integral term converges to 0 for a subsequence.

Since $|Z^{n}_{s}|$ and $\|U^{n}_{s}\|$ are uniformly integrable w.r.t. $\mathbb {P}\otimes \lambda,$ we imply from (29) that *χ*_*n*_→0 in $L^{1}(\mathbb {P}\otimes \lambda).$ Hence, there exists a subsequence (*n*_*k*_)_*k*≥1_ such that

31 $$\begin{array}{@{}rcl@{}}  \chi_{n_k} \to 0 \quad k\to\infty, \quad\mathbb{P}\otimes\lambda \text{-a.e.} \end{array} $$

By dominated convergence, we have $ \mathbb {E}\left | \int _{0}^{T}\chi _{n_{k}}(s) F(s)ds \right |^{2} \to 0 $ for *k*→*∞* since *F*∈*L*^2^(*Ω*;*L*^1^([0,*T*])).

For $ \delta ^{(2)}_{n,m},$ we start with the Cauchy–Schwarz inequality and get

$$\begin{array}{@{}rcl@{}} \delta^{(2)}_{n,m} &\le& 2 \sup_{k} \left [ \left\|Z^{k}\right\|_{L^{2}(W)} + \left\|U^{k}\right\|_{L^{2}(\tilde N) }\right] \\ &&\times \left[ \mathbb{E}\int_{0}^{T}\chi_{n}(s)\,K_{2}(s)^{2} \left(| Y^{n}_{s}|^{2} +|Y^{m}_{s}|^{2}\right)ds\right]^{\frac{1}{2}}. \end{array} $$

By Lemma 4.3,

32 $$ \begin{aligned} &\mathbb{E}\int_{0}^{T}\chi_{n}(s)\,K_{2}(s)^{2} \left(| Y^{n}_{s}|^{2} +|Y^{m}_{s}|^{2}\right)ds \\ &\le 2e^{2C_{K}} \mathbb{E}\int_{0}^{T}\chi_{n}(s)\,K_{2}(s)^{2}ds |\xi|^{2} \\ &+ 2e^{2C_K}\left\|\int_{0}^{T}\chi_{n}(s)\,K_{2}(s)^{2}ds \cdot I_{F} \right\|_{2}\left(\|Y^{n}\|_{\mathcal{S}^{2}}+ \|Y^{m}\|_{\mathcal{S}^{2}} \right). \end{aligned}  $$

Hence, (31) implies $\delta ^{(2)}_{n_{k},m} \to 0$ for *k*→*∞*.

Finally,

$$\begin{array}{@{}rcl@{}} \delta^{(3)}_{n,m} \le 2\mathbb{E}\int_{0}^{T} \left(2|Y^{n}_{s}|^{2} + |Y^{m}_{s}|^{2} \right)\, \chi_{n}(s) \,K_{1}(s)ds, \end{array} $$

so that we can argue like in (32) to get that $\delta ^{(3)}_{n_{k},m} \to 0$ for *k*→*∞*.

Thus $\phantom {\dot {i}\!}(Y^{n_{k}},Z^{n_{k}},U^{n_{k}})_{k\ge 1}$ converges to an object (*Y*,*Z*,*U*) in $L^{2}(W)\times L^{2}(W)\times L^{2}\left (\tilde {N}\right)$.

Step 4:

In the final step, we want to show that (*Y*,*Z*,*U*) solves (*ξ*,*f*). For the approximating sequence $\left (Y^{n_{k}},Z^{n_{k}},U^{n_{k}}\right)_{k\ge 1},$ the stochastic integrals and the left hand side of the BSDEs $\left (\xi,f^{(n_{k})}\right)$ obviously converge in *L*^2^ to the corresponding terms of (*ξ*,*f*). Therefore, this subsequence of $\left (\int _{t}^{T} f^{(n)}\left (s,Y^{n}_{s},Z^{n}_{s},U^{n}_{s}\right)ds\right)_{n=1}^{\infty }$ converges to a random variable *V*_*t*_. We need to show that $V_{t}=\int _{t}^{T} f(s,Y_{s},Z_{s},U_{s})ds$. To achieve this, consider

33 $$\begin{array}{@{}rcl@{}} \delta_{n}:=&\mathbb{E} \int_{t}^{T} |f^{(n)}\left(s,Y^{n}_{s},Z^{n}_{s},U^{n}_{s}\right) - f\left(s,Y^{n}_{s},Z^{n}_{s},U^{n}_{s}\right)|ds \\ &+ \mathbb{E}\int_{t}^{T}| f\left(s,Y^{n}_{s},Z^{n}_{s},U^{n}_{s}\right)-f\left(s,Y_{s},Z_{s},U_{s}\right)|ds. \end{array} $$

We start with the first integrand where, by the definition of *f*_*n*_ and (29), and the growth condition (A2),

$$\begin{array}{@{}rcl@{}} && |f^{(n)}\left(s,Y^{n}_{s},Z^{n}_{s},U^{n}_{s}\right) - f\left(s,Y^{n}_{s},Z^{n}_{s},U^{n}_{s}\right)| \\ &&\quad = |f^{(n)}\left(s,Y^{n}_{s},Z^{n}_{s},U^{n}_{s}\right) - f\left(s,Y^{n}_{s},Z^{n}_{s},U^{n}_{s}\right)| \chi_{n} \\ &&\quad \le 2\left(F(s) \chi_{n}(s) +K_{1}(s)|Y^{n}_{s}| \chi_{n}(s) +K_{2}(s)\chi_{n}(s)\left(|Z^{n}_{s}|+\|U^{n}_{s}\|\right)\right)\\ &&\quad =: 2\left(\kappa^{(1)}_{n}(s) +\kappa^{(2)}_{n}(s) +\kappa^{(3)}_{n}(s)\right). \end{array} $$

The estimates are similar as in the previous step. Thanks to (31), we have $\mathbb {E} \int _{t}^{T}\kappa ^{(1)}_{n_{k}}(s)ds \to 0.$ For the next term, the Cauchy–Schwarz inequality yields

$$\begin{array}{@{}rcl@{}} \mathbb{E} \int_{t}^{T}\kappa^{(2)}_{n_k}(s)ds \le \left \|\int_{0}^{T}\chi_{n}(s)\,K_{1}(s)ds \right\|_{2} \sup_{l} \|Y^{l}\|_{\mathcal{S}^{2}}, \end{array} $$

so that by (31) the first factor converges to zero along the subsequence (*n*_*k*_). The last term we estimate using the Cauchy–Schwarz inequality w.r.t. $\mathbb {P}\otimes \lambda,$

$$\begin{array}{@{}rcl@{}} \mathbb{E} \! \int_{t}^{T}\!\kappa^{(3)}_{n_k}(s)ds \le \left[ \mathbb{E} \int_{0}^{T}\!\!K_{2}(s)^{2}\chi_{n}(s) ds \right ]^{\frac{1}{2}} \sup_{l} \left [ \left\|Z^{l}\right\|_{L^{2}(W)} \!\!+ \left\|U^{l}\right\|_{L^{2}\left(\tilde{N}\right) }\right], \end{array} $$

and again by (31), we have convergence to zero along the subsequence (*n*_*k*_).

We continue showing the convergence of the second term in (33). We extract a sub-subsequence of (*n*_*k*_)_*k*≥1_, which we call—slightly abusing the notation—again (*n*_*k*_)_*k*≥1_ such that $\phantom {\dot {i}\!}(Y^{n_{k}},Z^{n_{k}},U^{n_{k}})$, regarded as a triplet of measurable functions with values in $\mathbb {R}\times \mathbb {R}\times L^{2}(\nu)$, converges to (*Y*,*Z*,*U*) for $\mathbb {P}\otimes \lambda $-a.a. (*ω*,*s*). Then, for an arbitrary *K*>0, we have

34 $$\begin{array}{@{}rcl@{}}  &\mathbb{E}\int_{t}^{T} \left|f\left(s,Y^{n_{k}}_{s},Z^{n_{k}}_{s},U^{n_{k}}_{s}\right)-f\left(s,Y_{s},Z_{s},U_{s}\right)\right|ds  \\ &\le \mathbb{E} \left\{\int_{t}^{T} \left|f\left(s,Y^{n_{k}}_{s},Z^{n_{k}}_{s},U^{n_{k}}_{s}\right)-f(s,Y_{s},Z_{s},U_{s})\right|\right.\\ &\quad\times\left.\left(\chi_{\left\{|Y^{n_{k}}_{s}|\leq K, |Z^{n_{k}}_{s}|+\|U^{n_{k}}_{s}\|\leq K\right\}}+\chi_{\left\{|Y^{n_{k}}_{s}|>K\right\}}+\chi_{\left\{ |Z^{n_{k}}_{s}|+\|U^{n_{k}}_{s}\|>K\right\}}\right)ds \right\}. \end{array} $$

By dominated convergence and the continuity of *f*,

$$\begin{array}{@{}rcl@{}} \mathbb{E}\!\int_{t}^{T} \!\!\left|f\left(s,Y^{n_{k}}_{s},Z^{n_{k}}_{s},U^{n_{k}}_{s}\right)-f(s,Y_{s},Z_{s},U_{s})\right|\!\chi\!_{\left\{|Y^{n_{k}}_{s}|\leq K, |Z^{n_{k}}_{s}|+\|U^{n_{k}}_{s}\|\leq K\right\}}ds \to 0, \end{array} $$

since by (A2) we can bound the integrand by

$$2F(s)+K_{1}(s)(K+|Y_{s}|)+K_{2}(s)(2K+|Z_{s}|+\|U_{s}\|), $$

which is integrable. We let

$$\chi_{K}(n_{k},s) := \chi_{\left\{|Y^{n_{k}}_{s}|> K\right\}}+\chi_{\left\{|Z^{n_{k}}_{s}|+\|U^{n_{k}}_{s}\| > K\right\}}. $$

Then, the remaining terms of (34) are bounded by

$$\begin{aligned} \mathbb{E}\int_{0}^{T}(2F(s)&+K_{1}(s)|Y_{s}|+K_{2}(s)(|Z_{s}|+\|U_{s}\|)) \chi_{K}(n_{k},s) ds \\ &+\mathbb{E}\int_{0}^{T}K_{1}(s)|Y^{n_{k}}_{s}| \chi_{K}(n_{k},s) ds \\ &+\mathbb{E}\int_{0}^{T} K_{2}(s)\left(|Z^{n_{k}}_{s}|+\|U^{n_{k}}_{s}\|\right)\chi_{K}(n_{k},s)ds \\ &=:\delta^{(1)}_{n_{k}} + \delta^{(2)}_{n_{k}}+ \delta^{(3)}_{n_{k}}. \end{aligned} $$

If we choose a *K* large enough, then $\delta ^{(1)}_{n_{k}}$ can be made arbitrarily small since the families $\left (|Y_{s}^{n}|,n\geq 0\right)$ and $\left (|Z^{n}_{s}|+\|U^{n}_{s}\|,n\geq 0\right)$ are uniformly integrable with respect to $\mathbb {P}\otimes \lambda $. The same holds for

$$\begin{array}{@{}rcl@{}} \left(\delta^{(2)}_{n_{k}}\right)^{2} &\le& \mathbb{E}\left|\int_{0}^{T}{K}_{1}(s)\chi_{K}(n_{k},s)ds\right|^{2} \,\sup_{l} \|Y^{n_{l}}\|_{\mathcal{S}^{2}}^{2}\\ &\le&\left\|\int_{0}^{T}K_{1}(s)ds\right\|_{\infty} \mathbb{E}\left[ \int_{0}^{T}K_{1}(s)\chi_{K}(n_{k},s)ds\right] \,\sup_{l} \|Y^{n_l}\|_{\mathcal{S}^{2}}^{2}, \end{array} $$

and

$$\begin{array}{@{}rcl@{}} \left(\delta^{(3)}_{n_k}\right)^{2} \le 2\mathbb{E}\left[\int_{0}^{T}K_{2}(s)^{2}\chi_{K}(n_{k},s)ds\right] \,\sup_{l} \mathbb{E}\int_{0}^{T}\left(\left|Z^{n_{l}}_{s}\right|^{2}+\left\|U^{n_{l}}_{s}\right\|^{2}\right)ds. \end{array} $$

Hence, for *δ*_*n*_ defined in (33), we have that ${\lim }_{k\to \infty } \delta _{n_{k}}=0,$ which implies

$${\lim}_{k\to\infty}\mathbb{E}\left|\int_{t}^{T} f^{(n_{k})}\left(s,Y^{n_{k}}_{s},Z^{n_{k}}_{s},U^{n_{k}}_{s}\right)ds-\int_{t}^{T} f(s,Y_{s},Z_{s},U_{s})ds\right|=0. $$

We infer that for a sub-subsequence $(n_{k_{l}},l\geq 0)$ we get the a.s. convergence

$$\int_{t}^{T} f^{(n_{k_{l}})}\left(s,Y^{n_{k_{l}}}_{s},Z^{n_{k_{l}}}_{s},U^{n_{k_{l}}}_{s}\right)ds\to\int_{t}^{T} f(s,Y_{s},Z_{s},U_{s})ds. $$

Thus, for the original sequence, a.s.

$$\int_{t}^{T} f^{(n_{k})}\left(s,Y^{n_{k}}_{s},Z^{n_{k}}_{s},U^{n_{k}}_{s}\right)ds\to V_{t}=\int_{t}^{T} f(s,Y_{s},Z_{s},U_{s})ds, $$

and therefore the triplet (*Y*,*Z*,*U*) satisfies the BSDE (*ξ*,*f*).

### Proof of Theorem 3.4

We start with a preparatory lemma:

#### **Lemma 5.1**

If *f* satisfies (A1)–(A4), then for all *n*≥0, *f*_*n*_ constructed in Definition 3.3 also satisfies (A1)–(A4) (with different coefficients).

#### *Proof*

By definition, (*ω*,*t*)↦*f*_*n*_(*t*,*y*,*z*,*u*) is progressively measurable for all (*y*,*z*,*u*), thus (A1) is satisfied. The inequalities in (A2) and (A3) are a.s. satisfied, with coefficients $\mathbb {E}_{n} F, \mathbb {E}_{n} K_{1}, \mathbb {E}_{n} K_{2}, \mathbb {E}_{n}\beta $. To ensure that these coefficients have a $\left (\mathcal {F}_{t}^{n}\right)_{t\in {[0,T]}}$-progressively measurable version, one applies the procedure from Definition 3.3 to the inequalities in (A2) and (A3) and notes that an equation analogous to (11) holds true.

It remains to show a.s. continuity of *f*_*n*_ in the (*y*,*z*,*u*)-variables required in (A3) for a.e. *t*. In (Ylinen (2017), Proposition 7.3), this was shown by the fact that the approximation of the generators appearing there can be done using spaces of continuous functions. However, since our situation involves *L*^2^(*ν*), a non-locally compact space, we can not easily adapt the proof from Ylinen (2017) and therefore we will use different means.

Let D[0,*T*] be the space of càdlàg functions endowed by the Skorohod metric (which makes this space a Polish space). The Borel *σ*-algebra $\mathcal {B}(\mathrm {D}[0,T])$ is generated by the coordinate projections $p_{t}\colon \mathrm {D}[0,T]\to \mathbb {R}, \mathrm {x}\mapsto \mathrm {x}(s)$ (see Theorem 12.5 of Billingsley (1968), for instance). On this *σ*-algebra, let $\mathbb {P}_{X}$ be the image measure induced by the Lévy process *X*: *Ω*→D[0,*T*],*ω*↦*X*(*ω*). We denote by $\mathcal {G}$ the completion with respect to $\mathbb {P}_{X}$. For *t*∈[0,*T*], the notation

$$\mathrm{x}^{t}(s):=\mathrm{x}(t\wedge s),\text{ for all} s\in{[0,T]}$$

induces the natural identification

$$\mathrm{D}{[0,t]}=\left\{\mathrm x\in \mathrm{D}{[0,T]} : \mathrm{x}^{t}=\mathrm{x} \right\}. $$

By this identification, we define a filtration on this space through

$$\begin{array}{@{}rcl@{}} \mathcal{G}_{t}=\sigma\left(\mathcal{B}\left(\mathrm{D}{[0,t]}\right)\cup \mathcal{N}_{X}{[0,T]}\right), \quad 0\leq t\leq T, \end{array} $$

where $\mathcal {N}_{X}{[0,T]}$ denotes the null sets of $\mathcal {B}\left (\mathrm {D}{[0,T]}\right)$ with respect to the image measure $\mathbb {P}_{X}$ of the Lévy process *X*. The same procedure applied to the Lévy process *X*^*n*^ yields a filtration $(\mathcal {G}_{t}^{n})_{t\in {[0,T]}}$ defined in the same way.

According to (Steinicke (2016), Theorem 3.4), which is a generalization of Doob’s factorization lemma to random variables depending on parameters, there is a $\mathcal {G}_{t}\otimes \mathcal {B}([0,t]\times \mathbb {R}^{2}\times L^{2}(\nu))$-measurable functional

$$g_{f}\colon \mathrm{D}[0,t]\times [0,t]\times\mathbb{R}^{2}\times L^{2}(\nu)\to\mathbb{R} $$

and a $\mathcal {G}_{t}^{n}\otimes \mathcal {B}([0, t]\times \mathbb {R}^{2}\times L^{2}(\nu))$-measurable functional

$$g_{f_{n}}\colon \mathrm{D}[0,t]\times [0, t]\times\mathbb{R}^{2}\times L^{2}(\nu)\to\mathbb{R} $$

such that $\mathbb {P}$-a.s.,

35 $$\begin{array}{@{}rcl@{}}  g_{f}(X(\omega),\cdot)=f(\omega,\cdot) \quad \text{and} \quad g_{f_n}(X^{n}(\omega),\cdot)=f_{n}(\omega,\cdot). \end{array} $$

Note also, that if $\mathbb {P}_{X}(M)=0$ for $M\in \mathcal {G}$, then also $\mathbb {P}(X^{-1}(M))=0$. Thus, without loss of generality, we may assume that $(\Omega,\mathcal {F},\mathbb {P})=(\mathrm {D}[0,T],\mathcal {G},\mathbb {P}_{X})$ and $(\Omega,\mathcal {F}_{t}^{n},\mathbb {P})=(\mathrm {D}([0,t]),\mathcal {G}_{t}^{n},\mathbb {P}_{X})$, which are standard Borel spaces. For more details on D[0,*T*], see Billingsley (1968) and (Delzeith (2004), Section 4).

Now, fix $N\in \mathbb {N}$ and let $c_{0}:=\{(a_{n})_{n}\in (\mathbb {R}^{2}\times L^{2}(\nu))^{\mathbb {N}}: a_{n}\to 0\}.$ For *a*∈*c*_0_, let $\|a\|_{c_{0}}=\sup _{n\in \mathbb {N}} (|a_{n}(1)|+|a_{n}(2)|+\|a_{n}(3)\|)$, where *a*(*k*),*k*=1,2,3 are the components of *a* in $\mathbb {R}$, $\mathbb {R}$ and *L*^2^(*ν*). The space *c*_0_ is a Polish space. Let *B*_*N*_ be the ball with radius $N\in \mathbb {N}$ in *c*_0_ and let $B^{\prime }_{N}$ be the ball of radius *N* in $\mathbb {R}^{2}\times L^{2}(\nu)$. The balls $B_{N}, B^{\prime }_{N}$ are again Polish spaces.

We consider a Borel set *M*_*T*_ of *t*∈[0,*T*] for which *f* is continuous in (*y*,*z*,*u*) and for which it holds that *f* has an integrable bound:

36 $$\begin{array}{@{}rcl@{}}  \mathbb{E} |f(t,y,z,u)| \le \mathbb{E} F(t) + \mathbb{E} K_{1}(t)|y|+ \mathbb{E} K_{2}(t)(|z|+\|u\|) < \infty. \end{array} $$

From (A3) and (9) it follows that one can choose *M*_*T*_ such that *λ*(*M*_*T*_)=*T*.

For a fixed *t*∈*M*_*T*_ we define the function

$$H_{m}:\Omega\times B_{N}\times B^{\prime}_{N}\to\mathbb{R}, \, (\omega,a,\varphi)\mapsto f_{n}(\omega,t,a_{m}+\varphi), $$

where *φ* denotes a triplet $(y,z,u)\in \mathbb {R}^{2}\times L^{2}(\nu)$. This function is measurable since *f*_*n*_(·,*t*,·) is measurable, $\pi _{m}:B_{N}\times B^{\prime }_{N}\to \mathbb {R}^{2}\times L^{2}(\nu), (a,\varphi)\mapsto (a_{m}+\varphi)$ is continuous and $\text {id}\times \pi _{m}:\Omega \times B_{N}\times B^{\prime }_{N}\to \Omega \times \mathbb {R}^{2}\times L^{2}(\nu)$ is measurable.

Next, we consider the map

$$ H:\Omega\times B_{N}\times B^{\prime}_{N}\to\mathbb{R}, (\omega,a,\varphi)\mapsto \begin{array}{ll} {\lim}_{m\to\infty}f_{n}(\omega,t,a_{m}+\varphi), & \quad \text{ if it exists}\\ 0, & \quad \text{else}. \end{array}  $$

The set, where the limit exists is measurable, since it can be written as

$$\bigcap_{k\geq 1}\bigcup_{M\geq 0}\bigcap_{m_{1},m_{2}\geq M}\left\{|H_{m_{1}}-H_{m_{2}}|<\frac{1}{k}\right\}. $$

Therefore, *H* can be written as the pointwise limit of measurable functions and is thus measurable.

We now know that, for a fixed pair $(a,\varphi)\in B_{N}\times B^{\prime }_{N}$,

$$f_{n}(t,a_{m}+\varphi)=\mathbb{E}_{n} f(t,a_{m}+\varphi), \quad\mathbb{P}\text{-a.s.} $$

Thus, by (36)

$$|f_{n}(t,a_{m}+\varphi)|\leq \mathbb{E}_{n} F(t)+2N\mathbb{E}_{n} K_{1}(t)+4N\mathbb{E}_{n} K_{2}(t) < \infty. $$

By the continuity of *f* and the dominated convergence theorem for conditional expectations, we infer that up to a null set $M(a,\varphi)\in \mathcal {F}^{n}_{t}$, we have the relation

$$\begin{array}{@{}rcl@{}} &{\lim}_{m\to\infty}f_{n}(t,a_{m}+\varphi)={\lim}_{m\to\infty}\mathbb{E}_{n} f(t,a_{m}+\varphi)=\mathbb{E}_{n} {\lim}_{m\to\infty} f(t,a_{m}+\varphi)\\ &=\mathbb{E}_{n} f(t,\varphi)=f_{n}(t,\varphi). \end{array} $$

In other words, on the complement of *M*(*a*,*φ*), we have *H*(*ω*,*a*,*φ*)=*f*_*n*_(*ω*,*t*,*φ*). This means that *H* and *f*_*n*_(·,*t*,·) are ”versions” of each other. What we need is ”indistinguishability” of the processes.

For this purpose, let $(A,\Phi):\Omega \to B_{N}\times B^{\prime }_{N}$ be an arbitrary $\mathcal {F}^{n}_{t}$-measurable function. Like above, by the definition of the optional projection, (A2), and the continuity of *f*, we get the equation

$$\begin{array}{@{}rcl@{}} &{\lim}_{m\to\infty}f_{n}(t,A_{m}+\Phi)=f_{n}(t,\Phi), \end{array} $$

which is also satisfied $\mathbb {P}$-a.s. This equality means, that

$$H(\omega,A(\omega),\Phi(\omega))= f_{n}(\omega, t,\Phi(\omega)),\quad a.s.$$

All $\mathcal {F}^{n}_{t}$ were complete *σ*-algebras (in fact they contain all null sets of $\mathcal {F}$) and the spaces $B_{N},B^{\prime }_{N}$ were Polish. Thus we may use a generalized version of the section theorem, the Jankov–von Neumann theorem (Theorem 5.3), by choosing a uniformizing function $\left (\hat A,\hat \Phi \right)$ for the set

$$P=\{(\omega,a,\varphi): H(\omega,a,\varphi)\neq f_{n}(\omega, t, \varphi)\}. $$

Note that *P* is a Borel set and therefore especially analytic, since *H* and *f*_*n*_(·,*t*,·) (interpreted as a constant map w.r.t. *a*) are measurable functions in (*ω*,*a*,*φ*). Since for this choice of $\left (\hat A,\hat \Phi \right)$ it holds, as seen above, that

$$H(\omega,\hat A(\omega),\hat \Phi(\omega))= f_{n}(\omega,t,\hat \Phi(\omega)), \quad \text{ a.s.} $$

it follows that the projection of *P* to *Ω* is a null set. Therefore, *H* and *f*_*n*_ are indistinguishable. Hence, we find a null set $M_{N}\in \mathcal {F}^{n}_{t}$, such that for *ω* outside this set and for all $(a,\varphi)\in B_{N}\times B^{\prime }_{N}$:

$${\lim}_{m\to\infty}f_{n}(\omega,t,a_{m}+\varphi)=f_{n}(\omega,t,\varphi). $$

But this means continuity in all points of $B^{\prime }_{N}$ a.s. It remains to unite the sets *M*_*N*_ for all $N\in \mathbb {N}$, to obtain a set such that on its complement the function is continuous in all points of $\mathbb {R}^{2}\times L^{2}(\nu)$. □

#### *Proof*

Step 1:

If *f* satisfies (A1)–(A4), by Lemma 5.1 all *f*_*n*_ do so as well. In this case, for all *n*≥0, the equations $(\mathbb {E}_{n}\xi,f_{n})$ have unique solutions by Theorem 3.1. In general, the coefficients in (A2) and *β* differ dependent on *n* since *F*,*K*_1_,*K*_2_,*β* will be replaced by the coefficients $\mathbb {E}_{n} F, \mathbb {E}_{n} K_{1}, \mathbb {E}_{n} K_{2}, \mathbb {E}_{n}\beta $.

Let us compare the solutions (*Y*^*n*^,*Z*^*n*^,*U*^*n*^) and (*Y*,*Z*,*U*). We start comparing (*Y*^*n*^,*Z*^*n*^,*U*^*n*^) and $(\mathbb {E}_{n} Y, \mathbb {E}_{n} Z, \mathbb {E}_{n} U)$. Here, for instance, the process $((\mathbb {E}_{n} Y)_{t})_{t\in {[0,T]}}$ is defined as an optional projection with respect to the filtration $\left (\mathcal {F}^{n}_{t}\right)_{t\in {[0,T]}}$, similar to Definition 3.3. The so defined processes are versions of the processes $(\mathbb {E}_{n} Y_{t},\mathbb {E}_{n} Z_{t}, \mathbb {E}_{n} U_{t})_{t\in {[0,T]}}$.

Using the BSDE for (*Y*,*Z*,*U*), we get $\mathbb {P}$-a.s.

37 $$\begin{array}{@{}rcl@{}} \mathbb{E}_{n} Y_{t}=& \,\mathbb{E}_{n} \xi+\int_{t}^{T}\mathbb{E}_{n} f(s,Y_{s},Z_{s},U_{s})ds - \int_{t}^{T}\mathbb{E}_{n} Z_{s} {dW}_{s}\\ &-\int_{{]t,T]}\times\{1/n \leq |x|\}}\mathbb{E}_{n} U_{s}(x)\tilde{N}(ds,dx), \end{array} $$

since

$$\mathbb{E}_{n} \int_{{]t,T]}\times\{1/n > |x|\}} U_{s}(x)\tilde{N}(ds,dx)=0. $$

Now, to estimate $\|Y^{n}-\mathbb {E}_{n} Y\|_{L^{2}(W)}+\|Z^{n}-\mathbb {E}_{n} Z\|_{L^{2}(W)}+\|U^{n}-\mathbb {E}_{n} U\|_{L^{2}(\tilde N)}$, we apply Itô’s formula to the difference of the BSDE $(\mathbb {E}_{n} \xi,f_{n})$ and (37). Similar to the proof of Proposition 4.2, we get, denoting differences by *Δ*^*n*^ and *η*:=4*β*(*s*)^2^,

$$\begin{array}{@{}rcl@{}} & \mathbb{E} \left\{e^{\int_{0}^{t}\eta(s)ds}|\Delta^{n} Y_{t}|^{2}+\int_{t}^{T} e^{\int_{0}^{s}\eta(\tau)d\tau}\left(\eta(s)|\Delta^{n} Y_{s}|^{2} +|\Delta^{n} Z_{s}|^{2}+ \|\Delta^{n} U_{s}\|^{2}\right)ds\right\} \\ &=\mathbb{E}\int_{t}^{T} 2e^{\int_{0}^{s}\eta(\tau)d\tau}(\Delta^{n} Y_{s}) \,\left(f_{n}\left(s,Y^{n}_{s},Z^{n}_{s},U^{n}_{s}\right)-\mathbb{E}_{n} f(s,Y_{s},Z_{s},U_{s})\right)ds. \end{array} $$

By the measurability of (*Y*^*n*^,*Z*^*n*^,*U*^*n*^), the equality

$$f_{n}\left(s,Y^{n}_{s},Z^{n}_{s},U^{n}_{s}\right)=\mathbb{E}_{n} f\left(s,Y^{n}_{s},Z^{n}_{s},U^{n}_{s}\right) $$

holds $\mathbb {P}$-a.s. for all *s*. We now estimate

$$\begin{array}{@{}rcl@{}} & \mathbb{E}\left[\left(\Delta^{n} Y_{s}\right) \,\left(f_{n}\left(s,Y^{n}_{s},Z^{n}_{s},U^{n}_{s}\right)-\mathbb{E}_{n} f\left(s,Y_{s},Z_{s},U_{s}\right)\right)\right]\\ = \,&\mathbb{E}\left[\left(\Delta^{n} Y_{s}\right) \,\left(\mathbb{E}_{n} f\left(s,Y^{n}_{s},Z^{n}_{s},U^{n}_{s}\right)-\mathbb{E}_{n} f\left(s,Y_{s},Z_{s},U_{s}\right)\right)\right]\\ = \,&\mathbb{E}\left[\left(\Delta^{n} Y_{s}\right) \,\left(f\left(s,Y^{n}_{s},Z^{n}_{s},U^{n}_{s}\right)-f\left(s,Y_{s},Z_{s},U_{s}\right)\right)\right]\\ =\,&\mathbb{E}\left[\left(\Delta^{n} Y_{s}\right) \,\left(f\left(s,Y^{n}_{s},Z^{n}_{s},U^{n}_{s}\right)-f\left(s,\mathbb{E}_{n} Y_{s},\mathbb{E}_{n} Z_{s},\mathbb{E}_{n} U_{s}\right)\right)\right]\\ &+\mathbb{E}\left[\left(\Delta^{n} Y_{s}\right) \,\left(f\left(s,\mathbb{E}_{n} Y_{s},\mathbb{E}_{n} Z_{s},\mathbb{E}_{n} U_{s}\right)-f\left(s,Y_{s},Z_{s},U_{s}\right)\right)\right]\\ \leq\,& \mathbb{E}\left[\alpha(s)\rho\left(|\Delta^{n} Y_{s}|^{2}\right)+\beta(s)|\Delta^{n} Y_{s}|\left(|\Delta^{n} Z_{s}|+\|\Delta^{n} U_{s}\|\right)\right]\\ &+\mathbb{E}\left[\left|\Delta Y^{n}_{s}\right|\left|\left(f\left(s,\mathbb{E}_{n} Y_{s},\mathbb{E}_{n} Z_{s},\mathbb{E}_{n} U_{s}\right)\right.-f\left(s,Y_{s},Z_{s},U_{s}\right)\right|\right]. \end{array} $$

Now, we can conduct exactly the same steps as in the standard procedure used in the proof of Proposition 4.2. This means that $\|\Delta ^{n} Y\|_{L^{2}(W)}+\|\Delta ^{n} Z\|_{L^{2}(W)} +\|\Delta ^{n} U\|_{L^{2}(\tilde N)}$ converges to zero if

38 $$\begin{array}{@{}rcl@{}} \mathbb{E}\int_{0}^{T} |\Delta Y^{n}_{s}||(f(s,\mathbb{E}_{n} Y_{s},\mathbb{E}_{n} Z_{s},\mathbb{E}_{n} U_{s})-f(s,Y_{s},Z_{s},U_{s})|ds \end{array} $$

does, which we will show in the following steps.

Step 2:

In this step, we show that the solution processes (*Y*^*n*^,*Z*^*n*^,*U*^*n*^) satisfy the estimate

39 $$ \sup_{n\geq 0} \left(\|Y^{n}\|_{\mathcal{S}^{2}} +\left\|Z^{n}\right\|_{L^{2}(W) }^{2} + \left\|U^{n}\right\|_{L^{2}(\tilde N) }^{2}\right)<\infty.  $$

This, as in the proof of Theorem 3.1, leads to the uniform integrability of the processes (|*Y*^*n*^|,*n*≥0) and (|*Z*^*n*^|+∥*U*^*n*^∥,*n*≥0) with respect to $\mathbb {P}\otimes \lambda $.

By Proposition 4.1, we get that

$$\begin{array}{@{}rcl@{}} &\|Y^{n}\|^{2}_{\mathcal{S}^{2}} + \left\|Z^{n}\right\|_{L^{2}(W) }^{2} + \left\|U^{n}\right\|_{L^{2}(\tilde N) }^{2} \leq e^{C_{1}(1+ C_{K,n})^{2}} \left(\mathbb{E} |\mathbb{E}_{n}\xi|^{2}+\mathbb{E} (I_{\mathbb{E}_{n}F})^{2}\right), \end{array} $$

where $C_{K,n}= \left \|\int _{0}^{T}\left (\mathbb {E}_{n} K_{1}(s)+(\mathbb {E}_{n} K_{2}(s))^{2}\right)ds\right \|_{\infty }$. By the monotonicity of $\mathbb {E}_{n}$ and Jensen’s inequality, we get that

$$\begin{array}{@{}rcl@{}} \int_{0}^{T}\!\!\left(\mathbb{E}_{n} K_{1}(s)+(\mathbb{E}_{n} K_{2}(s))^{2}\right)\!ds\leq\mathbb{E}_{n}\int_{0}^{T}\!\!\!\left(K_{1}(s)+K_{2}(s)^{2}\right)ds\leq C_{K},\,\,\mathbb{P}\text{-a.s.} \end{array} $$

Doob’s martingale inequality applied to $n\mapsto \mathbb {E}_{n}\xi $ and $n\mapsto I_{\mathbb {E}_{n}F}=\mathbb {E}_{n}\int _{0}^{T}F(s)ds$ yields that

$$\begin{array}{@{}rcl@{}} &\|Y^{n}\|^{2}_{\mathcal{S}^{2}} + \left\|Z^{n}\right\|_{L^{2}(W) }^{2} + \left\|U^{n}\right\|_{L^{2}(\tilde N) }^{2} \leq e^{C_{1}(1+ C_{K})^{2}} \left(\mathbb{E} |\xi|^{2}+\mathbb{E} (I_{F})^{2}\right). \end{array} $$

Furthermore,

40 $$ \sup_{n\geq 0} \left(\|\mathbb{E}_{n} Y\|_{\mathcal{S}^{2}} +\left\|\mathbb{E}_{n} Z\right\|_{L^{2}(W) }^{2} + \left\|\mathbb{E}_{n} U\right\|_{L^{2}\left(\tilde{N}\right) }^{2}\right)<\infty  $$

follows from martingale convergence and Jensen’s inequality and implies uniform integrability of the processes $\left ({|\mathbb {E}_{n} Y|},n\geq 0\right)\text { and }\left (|\mathbb {E}_{n} Z|+\|\mathbb {E}_{n} U\|,n\geq 0\right)$ with respect to $\mathbb {P}\otimes \lambda $.

Step 3:

In this step, we show the convergence (38). From martingale convergence, we get that for all *t*∈[0,*T*], $\mathbb {E}_{n} Y_{t}\to Y_{t}$, $\mathbb {E}_{n} Z_{t}\to Z_{t}$ and $\mathbb {E}_{n} U_{t}\to U_{t}$, $\mathbb {P}$-a.s. This implies that $f(s,\mathbb {E}_{n} Y_{s},\mathbb {E}_{n} Z_{s},\mathbb {E}_{n} U_{s})\to f(s,Y_{s},Z_{s},U_{s})$ in $\mathbb {P}\otimes \lambda $. Therefore,

$$\begin{array}{@{}rcl@{}} {\lim}_{n\to\infty}&\mathbb{E}\int_{0}^{T}|Y^{n}_{s}-\mathbb{E}_{n} Y_{s}||f(s,\mathbb{E}_{n} Y_{s},\mathbb{E}_{n} Z_{s},\mathbb{E}_{n} U_{s})- f(s,Y_{s},Z_{s},U_{s})|\\ &\quad\quad\times\chi_{\{|Y^{n}_{s}|+|\mathbb{E}_{n} Y_{s}|\leq K\}}ds=0 \end{array} $$

since the integrals form a uniformly integrable sequence with respect to $\mathbb {P}\otimes \lambda $. Indeed, we have, using (A2) for *f* and the first equation of (14), the estimate

$$\begin{array}{@{}rcl@{}} &|Y^{n}_{s}- \mathbb{E}_{n} Y_{s}||f\left(s,\mathbb{E}_{n} Y_{s},\mathbb{E}_{n} Z_{s},\mathbb{E}_{n} U_{s}\right)- f\left(s,Y_{s},Z_{s},U_{s}\right)|\chi_{\left\{|Y^{n}_{s}|+|\mathbb{E}_{n} Y_{s}|\leq K\right\}} \\ &\leq 4K (F(s)+K_{1}(s))\\ &\quad +2K\left(K_{2}(s)^{2}\right)+|Z_{s}|^{2}+\|U_{s}\|^{2}+|\mathbb{E}_{n} Z_{s}|^{2}+\|\mathbb{E}_{n} U_{s}\|^{2}), \end{array} $$

where $ n\mapsto \mathbb {E}_{n} Z_{s}, n\mapsto \mathbb {E}_{n} U_{s}$ converge since they are closable martingales.

Next, we will show that

41 $$\begin{array}{@{}rcl@{}} \delta_{n}(K):&= \mathbb{E} \left\{\int_{0}^{T}|Y^{n}_{s}-\mathbb{E}_{n} Y_{s}||f(s,\mathbb{E}_{n} Y_{s},\mathbb{E}_{n} Z_{s},\mathbb{E}_{n} U_{s})- f(s,Y_{s},Z_{s},U_{s})|\right.\\ &\times\left.\chi_{\{|Y^{n}_{s}|+|\mathbb{E}_{n} Y_{s}|> K\}}ds {\vphantom{\int_{0}^{T}}}\right\} \end{array} $$

can be made arbitrarily small by the choice of *K*>0, uniformly in *n*. Again by (A2) and using the notation $\chi ^{n}_{K}(s):= \chi _{\{|Y^{n}_{s}|+|\mathbb {E}_{n} Y_{s}|> K\}},$ we estimate like in (30)

$$\begin{array}{@{}rcl@{}} &|Y^{n}_{s}-\mathbb{E}_{n} Y_{s}||f\left(s,\mathbb{E}_{n} Y_{s},\mathbb{E}_{n} Z_{s},\mathbb{E}_{n} U_{s}\right)- f(s,Y_{s},Z_{s},U_{s})|\chi_{\left\{|Y^{n}_{s}|+|\mathbb{E}_{n} Y_{s}|> K\right\}} \\ &\le |Y^{n}_{s}- \mathbb{E}_{n} Y_{s}| \left(2F(s)+K_{1}(s)(|Y_{s}|+|\mathbb{E}_{n} Y_{s}|)\right.\\ &\quad\quad\quad\quad\quad\left.+K_{2}(s)(|Z_{s}|+|\mathbb{E}_{n} Z_{s}|+\|U_{s}\|+\|\mathbb{E}_{n} U_{s}\|)\right)\chi^{n}_{K}(s) \end{array} $$

and get

42 $$ {\begin{aligned} \delta_{n}(K) &\le 2\mathbb{E} \left\{\int_{0}^{T}\chi^{n}_{K}(s)\, F(s)ds \,\, \left(\sup_{r\in{[0,T]}}| Y^{n}_{r}|+\sup_{r\in{[0,T]}}|\mathbb{E}_{n} Y_{r}|\right) \right\}\\ &\quad + \mathbb{E} \left\{\int_{0}^{T} \chi^{n}_{K}(s)\,K_{2}(s)(| Y^{n}_{s}|+|\mathbb{E}_{n} Y_{s}|)(|Z_{s}|+|\mathbb{E}_{n} Z_{s}|+\|U_{s}\|+\|\mathbb{E}_{n} U_{s}\|)ds \right\}\\ &\quad +\mathbb{E}\int_{0}^{T} |Y^{n}_{s}-\mathbb{E}_{n} Y_{s}|\, (|Y_{s}|+|\mathbb{E}_{n} Y_{s}|) \chi^{n}_{K}(s) \,K_{1}(s)ds \\ &=: \delta^{(1)}_{n,K} + \delta^{(2)}_{n,K}+ \delta^{(3)}_{n,K}. \end{aligned}}  $$

For $\delta ^{(1)}_{n,K},$ we estimate

$$\delta^{(1)}_{n,K}\leq 2 \left\|\int_{0}^{T}\chi^{n}_{K}(s) F(s)ds\right\|_{2}\sup_{l\geq 0}\left(\|Y^{l}\|_{\mathcal{S}^{2}}+\|\mathbb{E}_{l} Y_{s}\|_{\mathcal{S}^{2}}\right) $$

which tends to zero as *K*→*∞*, since we have $\chi ^{n}_{K}\to 0$ in $\mathbb {P}\otimes \lambda $, uniformly in *n*, as *K*→*∞*. The latter is implied by the uniform integrability of the families (|*Y*^*n*^|)_*n*≥0_ and $(|\mathbb {E}_{n} Y|)_{n\geq 0}$ with respect to $\mathbb {P}\otimes \lambda.$ We continue with the next summands,

43 $$\begin{array}{@{}rcl@{}} &\delta^{(2)}_{n,K}\le 8\left(\mathbb{E}\int_{0}^{T}\left(| Y^{n}_{s}|^{2} +|\mathbb{E}_{n} Y_{s}|^{2}\right)\chi^{n}_{K}(s)\,K_{2}(s)^{2} ds\right)^{\frac{1}{2}}  \\ & \hspace{8em}\times\left (\left\|Z\right\|_{L^{2}(W)}+ \left\|U\right\|_{L^{2}\left(\tilde{N}\right) }\right) \end{array} $$

and

44 $$\begin{array}{@{}rcl@{}} &\delta^{(3)}_{n,K}\le \mathbb{E}\int_{0}^{T} \left(|Y_{s}|^{2}+|Y^{n}_{s}|^{2} + 2|\mathbb{E}_{n} Y_{s}|^{2}\right)\, \chi^{n}_{K}(s) \,K_{1}(s)ds, \end{array} $$

where, for $\mathbb {E}\int _{0}^{T}\chi ^{n}_{K}(s) \left (|Y_{s}|^{2} +|Y^{n}_{s}|^{2}\right)K_{1}(s)ds$ and $\mathbb {E}\int _{0}^{T} \chi ^{n}_{K}(s) |Y^{n}_{s}|^{2} K_{2}(s)^{2}ds,$ we will apply the estimate (27) from the proof of Lemma 4.3. For example (the other terms can be treated similarly), we get

45 $$ {\begin{aligned} \mathbb{E}\int_{0}^{T} \chi^{n}_{K}(s) | Y^{n}_{s}|^{2}\,K_{2}(s)^{2} ds &\leq \mathbb{E}\int_{0}^{T}\chi^{n}_{K}(s)\,K_{2}(s)^{2}ds\cdot e^{\int_{0}^{T} \eta_{n}(s)ds}|\mathbb{E}_{n}\xi|^{2}\\ & + 2\mathbb{E}\int_{0}^{T}\!\int_{0}^{s}\!\chi^{n}_{K}(s)\, K_{2}(\tau)^{2}d\tau\, e^{\int_{0}^{s} \eta_{n}(\tau)d\tau}\mathbb{E}_{n} F(s)|Y^{n}_{s}|ds \\ & \leq e^{2 C_{K}}\mathbb{E}\int_{0}^{T}\chi^{n}_{K}(s)\,K_{2}(s)^{2}ds|\mathbb{E}_{n} \xi|^{2}\\ & +2e^{2 C_{K}} \left\|\int_{0}^{T} \chi^{n}_{K}(s)\,K_{2}(s)^{2}ds\cdot I_{\mathbb{E}_{n} F}\right\|_{2}\|Y^{n}\|_{\mathcal{S}^{2}} \end{aligned}}  $$

with $\int _{0}^{T} \eta _{n}(s)ds =\int _{0}^{T} \mathbb {E}_{n} K_{1}(s)+(\mathbb {E}_{n} K_{2}(s))^{2}ds \le C_{K}$ a.s. Now, one gets that

$$\int_{0}^{T} \chi^{n}_{K}(s) K_{2}(s)^{2}ds \stackrel{\mathbb{P}}{\to} 0, \quad K\to \infty. $$

Furthermore, using $\sup _{n\geq 0}\mathbb {E}_{n}\int _{0}^{T}F(s)ds<\infty $, $\mathbb {P}$-a.s. (which follows from martingale convergence),

$$\int_{0}^{T} \chi^{n}_{K}(s) K_{2}(s)^{2}ds\int_{0}^{T}\mathbb{E}_{n}F(s)ds \stackrel{\mathbb{P}}{\to} 0, \quad K\to \infty, $$

independently of *n*. Since, by Doob’s maximal inequality,

$$\begin{array}{@{}rcl@{}} &\mathbb{E} \left[\sup_{n\geq 0}\int_{0}^{T}K_{2}(s)^{2}ds\int_{0}^{T}\mathbb{E}_{n}F(s)ds\right]^{2}\\ &\leq \mathbb{E} \left[\sup_{n\geq 0}C_{K} \mathbb{E}_{n}\int_{0}^{T} F(s) ds\right]^{2}\leq 4C_{K}^{2} \mathbb{E} I_{F}^{2}<\infty, \end{array} $$

dominated convergence is applicable to the last expression in (45). The first summand containing *ξ* can be treated in the same way.

The terms containing $|\mathbb {E}_{n} Y_{s}|$ in the inequalities (43) and (44), e.g., the expression $\mathbb {E}\int _{0}^{T} \chi ^{n}_{K}(s) |\mathbb {E}_{n} Y_{s}|^{2}K_{1}(s)ds,$ can be estimated by

$${\begin{aligned} \mathbb{E}\int_{0}^{T} \chi^{n}_{K}(s) |\mathbb{E}_{n} Y_{s}|^{2}K_{1}(s)ds&\leq \mathbb{E}\int_{0}^{T} \chi^{n}_{K}(s) K_{1}(s)\left(\sup_{l\geq 0}|\mathbb{E}_{l} Y_{s}|\right)^{2}ds\\ &\leq\mathbb{E} \left\{\int_{0}^{T}\chi^{n}_{K}(s) K_{1}(s)ds\left(\sup_{t\in{[0,T]}}\sup_{l\geq 0}\mathbb{E}_{l}|Y_{t}|\right)^{2} \right\}\\ &\leq 2C_{K}\|Y\|^{2}_{\mathcal{S}^{2}}, \end{aligned}} $$

where we used Doob’s maximal inequality again. Since $\int _{0}^{T}\chi ^{n}_{K}(s) K_{1}(s)ds\to 0$ in $\mathbb {P}$ as *K*→*∞*, all the terms in (43) and (44) become small, uniformly in *n*, if *K* is large. So the expressions $\delta ^{(2)}_{n,K}$ and $\delta ^{(3)}_{n,K}$ can be made arbitrarily small by the choice of *K*, which gives us the desired convergence

$$\mathbb{E}\int_{0}^{T} |Y^{n}_{s}-\mathbb{E}_{n} Y_{s}||f(s,\mathbb{E}_{n} Y_{s},\mathbb{E}_{n} Z_{s},\mathbb{E}_{n} U_{s})- f(s,Y_{s},Z_{s},U_{s})|ds\to 0. $$

Step 5:

Since, by the last step,

$$\|Y^{n}-\mathbb{E}_{n} Y\|_{L^{2}(W)}+\|Z^{n}-\mathbb{E}_{n} Z\|_{L^{2}(W)}+\|U^{n}-\mathbb{E}_{n} U\|_{L^{2}\left(\tilde{N}\right)}\to 0, $$

and also, by martingale convergence,

$$\|Y-\mathbb{E}_{n} Y\|_{L^{2}(W)}+\|Z-\mathbb{E}_{n} Z\|_{L^{2}(W)}+\|U-\mathbb{E}_{n} U\|_{L^{2}\left(\tilde{N}\right)}\to 0, $$

we get

$$\|Y^{n}- Y\|_{L^{2}(W)}+\|Z^{n}-Z\|_{L^{2}(W)}+\|U^{n}-U\|_{L^{2}\left(\tilde{N}\right)}\to 0. $$

□

## Appendix

**The Bihari–LaSalle inequality.** For the Bihari–LaSalle inequality we refer to (Mao (1997), pp. 45-46). Here, we formulate a backward version of it which has been applied in Yin and Mao (2008). The proof is analogous to that in Mao (1997).

### **Proposition 5.2**

Let *c*>0. Assume that *ρ*:[0,*∞*[→[0,*∞*[ is a continuous and non-decreasing function such that *ρ*(*x*)>0 for all *x*>0. Let *K* be a non-negative, integrable Borel function on [0,*T*], and *y* a non-negative, bounded Borel function on [0,*T*], such that

$$\begin{array}{@{}rcl@{}} y(t) &\le c + \int_{t}^{T} K(s) \rho(y(s)) ds. \end{array} $$

Then, it holds that

$$y(t) \le G^{-1} \left (G(c) + \int_{t}^{T} K(s) ds \right) $$

for all *t*∈[0,*T*] such that $G(c) + \int _{t}^{T} K(s) ds \in \text {dom}\left (G^{-1}\right).$ Here

$$\begin{array}{@{}rcl@{}} G(x) := \int_{1}^{x} \frac{dr}{\rho(r)}, \end{array} $$

and *G*^−1^ is the inverse function of *G*.Especially, if *ρ*(*r*)=*r* for *r*∈[0,*∞*[, it holds that

46 $$\begin{array}{@{}rcl@{}}  y(t)\le c e^{\int_{t}^{T} K(s) ds}. \end{array} $$

**The Jankov–von Neumann theorem.** If *X* and *Y* are sets and *P*⊆*X*×*Y*, then *P*^∗^⊆*P* is called a *uniformization* of *P* if and only if *P*^∗^ is the graph of a function *f*:proj_*X*_(*P*)→*Y*, i.e., *P*^∗^={(*x*,*f*(*x*)):*x*∈proj_*X*_(*P*)}. Such a function *f* is called a *uniformizing function* for *P*. Let $ \Sigma _{1}^{1}(X)$ denote the class of analytic subsets of *X*. The following theorem can be found, for example, in (Kechris (1994), Theorem 18.1).

### **Theorem 5.3**

(Jankov–von Neumann theorem) Assume that *X* and *Y* are standard Borel spaces and *P*⊆*X*×*Y* is an analytic set. Then, *P* has a uniformizing function that is $\sigma \left (\Sigma _{1}^{1}(X)\right)$- measurable.
